# Supplementary material for: Effects of Extracted Pulse Proteins on Lipid Targets for Cardiovascular Risk Reduction: Systematic Review and Meta-Analysis of Randomized Controlled Trials
Source: Nutrients. 2024 Nov 1;16(21):3765. doi: 10.3390/nu16213765 (PMC11548137; doi:10.3390/nu16213765)
Supplement: Supplementary file 1 [file nutrients-16-03765-s001.zip › nutrients-3266623-supplementary.pdf]

Supplemental Materials

Re: Yang et al. Effects of Extracted Pulse Proteins on Lipid Targets for Cardiovascular Risk Reduction: A Systematic Review and Meta-analysis of Randomized Controlled Trials

Table of Contents

Supplemental Table S1. PRISMA Checklist 5

Supplemental Table S2. Search strategy for randomized controlled trials assessing the effect of extracted pulse proteins on blood lipids........ 8

Supplemental Table S3. PICOTS framework of the search strategy........ 11

Supplemental Table S4. Table of characteristics of the randomized controlled trial assessing the effect of extracted pulse proteins on blood lipids 12

Supplemental Table S5. Assessment of study product acceptability and adverse events* 14

Supplemental Table S6. Sensitivity analyses of the use of correlation coefficients of 0.25 and 0.75 for crossover trials in the primary analysis of the effect of extracted pulse proteins on blood lipids........ 15

Supplemental Table S7. GRADE assessment of the certainty of evidence........ 16

Supplemental Figure S1. Risk of bias proportion graph for the effect of extracted pulse proteins on blood lipids in parallel trials 17

Supplemental Figure S2. Risk of bias proportion graph for the effect of extracted pulse proteins on blood lipids in crossover trials 19

Supplemental Figure S3. Forest plot of randomized controlled trials of the effect of extracted pulse proteins on LDL-C 21

Supplemental Figure S4. Forest plot of randomized controlled trials of the effect of extracted pulse proteins on non-HDL-C........ 22

Supplemental Figure S5. Forest plot of randomized controlled trials of the effect of extracted pulse proteins on HDL-C 23

Supplemental Figure S6. Forest plot of randomized controlled trials of the effect of extracted pulse proteins on TG 24

Supplemental Figure S7. Sensitivity analysis of the systematic removal of each trial for the effect of extracted pulse proteins on LDL-C 25

Supplemental Figure S8. Sensitivity analysis of the systematic removal of each trial for the effect of extracted pulse proteins on non-HDL-C........ 26

Supplemental Figure S9. Sensitivity analysis of the systematic removal of each trial for the effect of extracted pulse proteins on HDL-C 27

Supplemental Figure S10. Sensitivity analysis of the systematic removal of each trial for the effect of extracted pulse proteins on TG 28

Supplemental Figure S11 (1 of 3). Subgroup analyses for the effect of extracted pulse proteins on LDL-C........ 29

Supplemental Figure S11 (2 of 3). Subgroup analyses for the effect of extracted pulse proteins on LDL-C........ 30

Supplemental Figure S11 (3 of 3). Subgroup analyses for the effect of extracted pulse proteins on LDL-C 31

Supplemental Figure S12 (1 of 3). Subgroup analyses for the effect of extracted pulse proteins on non-HDL-C 32

Supplemental Figure S12 (2 of 3). Subgroup analyses for the effect of extracted pulse proteins on non-HDL-C 33

Supplemental Figure S12 (3 of 3). Subgroup analyses for the effect of extracted pulse proteins on non-HDL-C 34

Supplemental Figure S13 (1 of 3). Subgroup analyses for the effect of extracted pulse proteins on HDL-C 35

Supplemental Figure S13 (2 of 3). Subgroup analyses for the effect of extracted pulse proteins on HDL-C 36

Supplemental Figure S13 (3 of 3). Subgroup analyses for the effect of extracted pulse proteins on HDL-C 37

Supplemental Figure S14 (1 of 3). Subgroup analyses for the effect of extracted pulse proteins on TG 38

Supplemental Figure S14 (2 of 3). Subgroup analyses for the effect of extracted pulse proteins on TG 39

Supplemental Figure S14 (3 of 3). Subgroup analyses for the effect of extracted pulse proteins on TG 40

Supplemental Figure S15. Risk of bias subgroup analyses for the effect of extracted pulse proteins on LDL-C 41

Supplemental Figure S16. Risk of bias subgroup analyses for the effect of extracted pulse proteins on non-HDL-C 42

Supplemental Figure S17. Risk of bias subgroup analyses for the effect of extracted pulse proteins on HDL-C 43

Supplemental Figure S18. Risk of bias subgroup analyses for the effect of extracted pulse proteins on TG 44

Supplemental Figure S19. Continuous meta-regression analysis for the effect of extracted pulse proteins on LDL-C* 45

Supplemental Figure S20. Continuous meta-regression analysis for the effect of extracted pulse proteins on non-HDL-C* 46

Supplemental Figure S21. Continuous meta-regression analysis for the effect of extracted pulse proteins on HDL-C* 47

Supplemental Figure S22. Continuous meta-regression analysis for the effect of extracted pulse proteins on TG* 48

Supplemental Figure S23. Continuous meta-regression analysis for the effect of extracted pulse proteins on LDL-C* (with Frota et al. 2015 removed) 49

Supplemental Figure S24. Continuous meta-regression analysis for the effect of extracted pulse proteins on non-HDL-C* (with Frota et al. 2015 removed) 50

Supplemental Figure S25. Linear and non-linear meta-regression analysis for the effect of extracted pulse proteins on LDL-C 51

Supplemental Figure S26. Linear and non-linear meta-regression analysis for the effect of extracted pulse proteins on non-HDL-C 52

Supplemental Figure S27. Linear and non-linear meta-regression analysis for the effect of extracted pulse proteins on HDL-C 53

Supplemental Figure S28. Linear and non-linear meta-regression analysis for the effect of extracted pulse proteins on TG 54

Supplemental Figure S29. Publication bias funnel plots for the effect of extracted pulse proteins on LDL-C 55

Supplemental Figure S30. Publication bias funnel plots for the effect of extracted pulse proteins on non-HDL-C 56

Supplemental Figure S31. Publication bias funnel plots for the effect of extracted pulse proteins on HDL-C 57

Supplemental Figure S32. Publication bias funnel plots for the effect of extracted pulse proteins on TG 58

Supplemental Table S1. PRISMA Checklist

Section and Topic	Item #	Checklist item	Location where item is reported
**TITLE**			
Title	1	Identify the report as a systematic review.	Page 1, 2
**ABSTRACT**			
Abstract	2	See the PRISMA 2020 for Abstracts checklist.	Page 1-2
**INTRODUCTION**			
Rationale	3	Describe the rationale for the review in the context of existing knowledge.	Page 2
Objectives	4	Provide an explicit statement of the objective(s) or question(s) the review addresses.	Page 2
**METHODS**			
Eligibility criteria	5	Specify the inclusion and exclusion criteria for the review and how studies were grouped for the syntheses.	Page 3<br>Supplemental Table A3
Information sources	6	Specify all databases, registers, websites, organisations, reference lists and other sources searched or consulted to identify studies. Specify the date when each source was last searched or consulted.	Page 3
Search strategy	7	Present the full search strategies for all databases, registers and websites, including any filters and limits used.	Supplemental Table A2
Selection process	8	Specify the methods used to decide whether a study met the inclusion criteria of the review, including how many reviewers screened each record and each report retrieved, whether they worked independently, and if applicable, details of automation tools used in the process.	Page 3
Data collection process	9	Specify the methods used to collect data from reports, including how many reviewers collected data from each report, whether they worked independently, any processes for obtaining or confirming data from study investigators, and if applicable, details of automation tools used in the process.	Page 3
Data items	10a	List and define all outcomes for which data were sought. Specify whether all results that were compatible with each outcome domain in each study were sought (e.g. for all measures, time points, analyses), and if not, the methods used to decide which results to collect.	Page 3
	10b	List and define all other variables for which data were sought (e.g. participant and intervention characteristics, funding sources). Describe any assumptions made about any missing or unclear information.	Page 3-4
Study risk of bias assessment	11	Specify the methods used to assess risk of bias in the included studies, including details of the tool(s) used, how many reviewers assessed each study and whether they worked independently, and if applicable, details of automation tools used in the process.	Page 3
Effect measures	12	Specify for each outcome the effect measure(s) (e.g. risk ratio, mean difference) used in the synthesis or presentation of results.	Page 3-4
Synthesis methods	13a	Describe the processes used to decide which studies were eligible for each synthesis (e.g. tabulating the study intervention characteristics and comparing against the planned groups for each synthesis (item #5)).	
	13b	Describe any methods required to prepare the data for presentation or synthesis, such as handling of missing summary statistics, or data conversions.	Page 3-4
	13c	Describe any methods used to tabulate or visually display results of individual studies and syntheses.	Page 4<br>Supplemental Table A4
	13d	Describe any methods used to synthesize results and provide a rationale for the choice(s). If meta-analysis was performed, describe the model(s), method(s) to identify the presence and extent of statistical heterogeneity, and software package(s) used.	Page 4
	13e	Describe any methods used to explore possible causes of heterogeneity among study results (e.g. subgroup analysis, meta-regression).	Page 4
	13f	Describe any sensitivity analyses conducted to assess robustness of the synthesized results.	Page 4

Reporting bias assessment	14	Describe any methods used to assess risk of bias due to missing results in a synthesis (arising from reporting biases).	Page 4
Certainty assessment	15	Describe any methods used to assess certainty (or confidence) in the body of evidence for an outcome.	Page 5
**RESULTS**			
Study selection	16a	Describe the results of the search and selection process, from the number of records identified in the search to the number of studies included in the review, ideally using a flow diagram.	Page 5<br>Figure 1
	16b	Cite studies that might appear to meet the inclusion criteria, but which were excluded, and explain why they were excluded.	Figure 1
Study characteristics	17	Cite each included study and present its characteristics.	Page 5-8<br>Supplemental Table A4
Risk of bias in studies	18	Present assessments of risk of bias for each included study.	Page 9<br>Supplemental Figure A1-A2
Results of individual studies	19	For all outcomes, present, for each study: (a) summary statistics for each group (where appropriate) and (b) an effect estimate and its precision (e.g. confidence/credible interval), ideally using structured tables or plots.	Page 9<br>Figure 2<br>Supplemental Figure A3-A6
Results of syntheses	20a	For each synthesis, briefly summarise the characteristics and risk of bias among contributing studies.	Page 9
	20b	Present results of all statistical syntheses conducted. If meta-analysis was done, present for each the summary estimate and its precision (e.g. confidence/credible interval) and measures of statistical heterogeneity. If comparing groups, describe the direction of the effect.	Page 9<br>Supplemental Figure A3-A6
	20c	Present results of all investigations of possible causes of heterogeneity among study results.	Page 10-11<br>Supplemental Figure A7-A24
	20d	Present results of all sensitivity analyses conducted to assess the robustness of the synthesized results.	Page 10<br>Supplemental Table A6 & Figure A7-A10
Reporting biases	21	Present assessments of risk of bias due to missing results (arising from reporting biases) for each synthesis assessed.	Page 9<br>Supplemental Figure A1-A2
Certainty of evidence	22	Present assessments of certainty (or confidence) in the body of evidence for each outcome assessed.	Page 11<br>Figure 2<br>Supplemental Table A6
**DISCUSSION**			

Discussion	23a	Provide a general interpretation of the results in the context of other evidence.	Page 11
	23b	Discuss any limitations of the evidence included in the review.	Page 13
	23c	Discuss any limitations of the review processes used.	Page 13
	23d	Discuss implications of the results for practice, policy, and future research.	Page 13-14
**OTHER INFORMATION**			
Registration and protocol	24a	Provide registration information for the review, including register name and registration number, or state that the review was not registered.	Page 3
	24b	Indicate where the review protocol can be accessed, or state that a protocol was not prepared.	Page 3
	24c	Describe and explain any amendments to information provided at registration or in the protocol.	N/A
Support	25	Describe sources of financial or non-financial support for the review, and the role of the funders or sponsors in the review.	Page 14-15
Competing interests	26	Declare any competing interests of review authors.	Page 15-16
Availability of data, code and other materials	27	Report which of the following are publicly available and where they can be found: template data collection forms; data extracted from included studies; data used for all analyses; analytic code; any other materials used in the review.	Page 15

From: Page MJ, McKenzie JE, Bossuyt PM, Boutron I, Hoffmann TC, Mulrow CD, et al. The PRISMA 2020 statement: an updated guideline for reporting systematic reviews. BMJ 2021;372:n71. doi: 10.1136/bmj.n71

Supplemental Table S2. Search strategy for randomized controlled trials assessing the effect of extracted pulse proteins on blood lipids

	MEDLINE		EMBASE		COCHRANE register for controlled trials
**1**	Fabaceae.mp. or exp Fabaceae/	1	Fabaceae/	1	Fabaceae.mp. or Fabaceae/
2	lentil*.mp.	2	Fabaceae.mp.	2	lentil*.mp.
3	chickpea*.mp.	3	lentil*.mp.	3	lentil/
4	bean*.mp.	4	exp lentil/	4	chickpea*.mp.
5	legume*.mp.	5	chickpea*.mp.	5	exp chickpea/
6	leguminous.mp.	6	exp chickpea/	6	bean*.mp.
7	Lupinus/ or lupin.mp.	7	bean*.mp.	7	legume*.mp.
8	betches.mp.	8	exp bean/	8	leguminous.mp.
9	"bambara ground nut*".mp.	9	legume*.mp.	9	lupin.mp.
10	"bambara groundnut*".mp.	10	exp legume/	10	exp Lupinus/
11	mung.mp.	11	leguminous.mp	11	betches.mp.
12	lens culinaris.mp. or Lens Plant/	12	lupin.mp. or exp lupin/	12	"bambara ground nut*".mp.
13	cicer arietinum.mp. or Cicer/	13	betches.mp.	13	"bambara groundnut*".mp.
14	garbanzo.mp.	14	"bambara ground nut*".mp.	14	mung.mp.
15	phaseolus vulgaris.mp. or Phaseolus/	15	"bambara groundnut*".mp.	15	lens culinaris.mp.
16	Peas/ or pea*.mp.	16	exp mung bean/ or mung.mp.	16	cicer arietinum.mp.
17	Lupinus/ or lupin*.mp.	17	lens culinaris.mp.	17	garbanzo.mp. or Cicer/
18	Black matpe*.mp.	18	cicer arietinum.mp.	18	exp Phaseolus vulgaris/
19	guar.mp.	19	garbanzo.mp.	19	Phaseolus vulgaris.mp. or exp Phaseolus/
20	carob.mp.	20	phaseolus vulgaris.mp.	20	cowpea*.mp.
21	vetch*.mp.	21	exp Phaseolus vulgaris/	21	triglyceride.mp.
22	lablab.mp.	22	exp cowpea/ or cowpea*.mp.	22	triacylglycerol.mp.
23	alfalfa.mp.	23	"meat alternative*".mp.	23	VLDL.mp.
24	horse gram.mp.	24	(meat adj8 alternative*).mp.	24	very low density lipoprotein.mp.
25	Macrotyloma uniflorum.mp.	25	exp meat substitute/	25	lipid*.mp.
26	cajanus/	26	meat substitute*.mp.	26	cholesterol.mp.
27	chamaecrista/	27	exp lipoproteins/	27	lipoprotein.mp.
28	lotus/	28	exp cholesterol/	28	(hdl or high density lipoprotein).mp.
29	mucuna/	29	exp hyperlipidemias/	29	(ldl or low density lipoprotein).mp.
30	cajanus.mp.	30	(lipid or lipids).mp.	30	hyperlipidemia*.mp.
31	chamaecrista.mp.	31	(cholesterol or cholesterols).mp.	31	apolipoprotein*.mp.
32	lotus.mp.	32	hdl.mp.	32	meat alternative*.mp.
33	mucuna.mp.	33	("high density lipoprotein" or "high density lipoproteins").mp.	33	(meat adj8 alternative*).mp.
34	phaseolus/	34	ldl.mp.	34	meat substitute*.mp.
35	phaseolus.mp.	35	("low density lipoprotein" or "low density lipoproteins").mp.	35	(or/1-20) or (or/32-34)
36	sphenostylis/	36	apolipoprotein*.mp.	36	or/21-31

37	**sphenostylis.mp.**	37	**(hyperlipemia* or hyperlipaemia*).mp.**	37	**35 and 36**
38	exp vicia/	38	(hyperlipidemia* or hyperlipidaemia*).mp.		
39	exp cowpea/ or cowpea*.mp.	39	(lipidemia* or lipidaemia*).mp.		
40	"meat alternative*".mp.	40	(lipemia* or lipaemia*).mp.		
41	(meat adj8 alternative*).mp.	41	(lipemic or lipaemic).mp.		
42	meat substitute*.mp.	42	or/1-26		
43	exp lipoproteins/	43	or/27-41		
44	exp cholesterol/	44	and/42-43		
45	exp hyperlipidemias/	45	randomized controlled trial'/		
46	(lipid or lipids).mp.	46	controlled clinical trial'/		
47	(cholesterol or cholesterols).mp.	47	random*'.ti,ab,tt.		
48	hdl.mp.	48	randomization'/		
49	("high density lipoprotein" or "high density lipoproteins").mp.	49	placebo.ti,ab,tt.		
50	ldl.mp.	50	(compare or compared or comparison).ti,tt.		
51	("low density lipoprotein" or "low density lipoproteins").mp.	51	(evaluated or evaluate or evaluating or assessed or assess).ab.		
52	apolipoprotein*.mp.	52	(compare or compared or comparing or comparison).ab.		
53	(hyperlipemia* or hyperlipaemia*).mp.	53	51 and 52		
54	(hyperlipidemia* or hyperlipidaemia*).mp.	54	(open adj label).ti,ab,tt.		
55	(lipidemia* or lipidaemia*).mp.	55	((double or single or doubly or singly) adj (blind or blinded or blindly)).ti,ab,tt.		
56	(lipemia* or lipaemia*).mp.	56	double blind procedure/		
57	(lipemic or lipaemic).mp.	57	(parallel adj group*).ti,ab,tt.		
58	or/1-42	58	(crossover or "cross over").ti,ab,tt.		
59	or/43-57	59	((assign* or match or matched or allocation) adj6 (alternate or group or groups or intervention or interventions or patient or patients or subject or subjects or participant or participants)).ti,ab,tt.		
60	and/58-59	60	(assigned or allocated).ti,ab,tt.		
61	randomized controlled trial.pt.	61	(controlled adj8 (study or design or trial)).ti,ab,tt.		
62	controlled clinical trial.pt.	62	(volunteer or volunteers).ti,ab,tt.		
63	randomized.ab.	63	human experiment/		
64	placebo.ab.	64	trial.ti,tt.		
65	clinical trials as topic.sh.	65	(or/45-50) or (or/53-64)		
66	randomly.ab.	66	(random* adj sampl* adj8 ("cross section*" or questionnaire* or survey or surveys or database or databases)).ti,ab,tt. not (comparative study/ or controlled study/ or "randomised controlled".ti,ab,tt. or "randomized controlled".ti,ab,tt. or "randomly assigned".ti,ab,tt.)		

68	**or/61-67**	68	**(("case control*" and random*) not ("randomised controlled" or "randomized controlled")).ti,ab,tt.**
69	exp animals/ not humans.sh.	69	("systematic review" not (trial or study)).ti,tt.
70	68 not 69	70	(nonrandom* not random*).ti,ab,tt.
71	60 and 70	71	"random field*".ti,ab,tt.
		72	("random cluster" adj4 sampl*).ti,ab,tt.
		73	(review.ab. and review.pt.) not trial.ti,tt.
		74	"we searched".ab. and (review.ti,tt. or review.pt.)
		75	"update review".ab.
		76	(databases adj5 searched).ab.
		77	(rat or rats or mouse or mice or swine or porcine or murine or sheep or lambs or pigs or piglets or rabbit or rabbits or cat or cats or dog or dogs or cattle or bovine or monkey or monkeys or trout or marmoset*).ti,tt. and animal experiment/
		78	animal experiment/ not (human experiment/ or human/)
		79	or/66-78
		80	65 not 79
		81	80 and 44

Supplemental Table S3. PICOTS framework of the search strategy

Participants	Interventions	Comparators	Outcomes	Time	Study design
Adult individuals of all health backgrounds	Dietary interventions of (mostly) extracted pulse proteins, including beans, chickpeas, peas and lentils or mixed pulse/beans diets, not including peanuts or soybeans	Suitable non-pulse, non-soy or non-peanut containing control	LDL-C, non-HDL-C, apoB, HDL-C, and TG, mean difference and 95% confidence intervals	≥3 weeks	Randomized controlled trials in humans

apoB, apolipoprotein B; HDL-C, high-density lipoprotein cholesterol; LDL-C, low-density lipoprotein cholesterol; non-HDL-C, non-high density lipoprotein cholesterol; PICOTS, participants, interventions, comparators, outcomes, time, and study design; TG, triglyceride

Supplemental Table S4. Table of characteristics of the randomized controlled trial assessing the effect of extracted pulse proteins on blood lipids

			Baseline																							
Study, Year	Intervention, Control	Participants number (M, F); health status	Gender info (Y/NR)	Mean Age, years (SD/Range)	Setting (IP/OP)	Country	BW (kg) (SD/range)	BMI (kg/m^2) (SD/range)	Mean LDL ± SD/range (mmol/L)	Mean HDL, mmol/L (SD/range)	Mean TG, mmol/L (SD/range)	Mean non-HDL, mmol/L (SD/range)	Mean ApoB, g/L (SD/range)	Design, washout length	Feeding Control[a]	Intervention or Comparator	Intervention and Food Source	Pulse protein dose (g/d)	Diet (% C:F:P)	SFA content (g/d; %E)	Fiber content (g/d; %E)	Energy Balance[b]	Energy Control[c]	Follow-up	Funding Sources[d]	Lipid Medication use (Y/N)
**Beans**																										
		43 Hypercholesterolaemic (20M, 23F)	NR		OP	Germany							NR	P	Supp				NR	NR	NR	Neutral	Substitution	6 wks	I	N
Weiße et al. 2010	Intervention	22 Hypercholesterolaemic (11M, 11F)		44.4 (12.2)			77.4 (17.2)	26.2 (5.0)	3.6 (0.7)	1.75 (0.48)	1.24 (0.5)	3.91 (0.8)				Lupin protein	Extracted protein incorporated into a snack bar along with wheat flour, honey, baking powder and hazelnut flavour	35								
	Control	21 Hypercholesterolaemic (9M, 12F)		43.3 (11.8)			76.3 (13.2)	25.7 (4.0)	3.7 (0.6)	1.70 (0.38)	1.59 (1.0)	4.08 (0.9)				Milk protein										
		47 Hypercholesterolaemic	NR		OP	Italy	NR						NR	P	Supp				54:24:17	9.1 (5.1)	33.8 (8.4)	Neutral	Substitution	4 wks	A	N
Sirtori et al. 2011 (lupin+cellulose)	Intervention	22 Hypercholesterolaemic		52.3 (12.4)				24.0 (2.0)	4.9 (0.9)	1.4 (0.4)	1.6 (0.8)	5.7 (1.1)				Lupin protein + cellulose	Extracted protein into a snack bar	34.6								
	Control	25 Hypercholesterolaemic		54.7 (10.5)				25.4 (4.2)	4.9 (0.9)	1.5 (0.3)	1.4 (0.5)	5.5 (0.9)				Milk protein + cellulose										
		68 Hypercholesterolaemic (28M, 40F)	NR	56.9 (10.7)	OP	Germany	76.9 (15.8)	26.5 (5.0)	4.1 (0.9)	1.4 (0.4)	1.8 (1.5)	4.9 (1.1)	NR	C (6 wks)	Supp					NR		Neutral	Substitution	28 d	A	N
	Intervention															Lupin protein	Extracted protein incorporated into bread, rolls, scalded sausage, vegetarian spread	25	48:35:16		37.4 (6.3)					
Bähr et al. 2015	Control (milk protein)															Milk protein			47:35:16		36.6 (6.1)					
	Control (milk protein with arg)															Milk protein with arginine			47:35:14		36.6 (6.1)					
		38 Hypercholesterolaemic (6M, 32F)	NR	57.0 (10.5)	OP	Brazil	66.7 (14.2)	27.3 (3.7)	4.7 (2.5)	1.5 (0.3)	1.8 (0.7)	5.5 (0.7)	1.3 (0.03)	C (4 wks)	Supp							Neutral	Substitution	6 wks	I	N
Frota et al. 2015	Intervention															Cowpea protein	Extracted cowpea protein in the form of ready-to-drink shake	25	52:22:26	8.2 (5.3)	11.7 (3.3)					
	Control															Milk protein			50:23:26	8.3 (5.5)	11.8 (3.4)					
		44 Absence of disease (19M, 25F)	NR		OP	USA & Canada							NR	P	Supp				NR	NR	NR	Positive	Substitution	8 wks	A+I	N
Kohno et al. 2018 (main study)	Intervention	22 Absence of disease (10M, 12F)		41.6 (1.8)			86.5 (10.3)	30.5 (3)	3.2 (0.9)	1.3 (0.3)	1.7 (1.9)	3.8 (1.0)				Mung bean protein tablet	Extracted mung bean protein + casein protein made into a chewable tablet	5.3								
	Control	22 Absence of disease (9M, 13F)		42.8 (1.8)			92.4 (11.7)	30.7 (2.9)	3.1 (0.5)	1.3 (0.2)	1.5 (0.5)	3.8 (0.6)				Milk protein tablet	Casein protein chewable tablet									
			NR		OP	USA & Canada			NR	NR		NR	NR	P	Supp				NR	NR	NR	Positive	Substitution	4 wks	A+I	N
	Intervention	7 Absence of disease (5M, 2F)		40.0 (13.7)			88.8 (18.8)	29.1 (3.8)			1.2 (0.4)					Mung bean protein tablet	Extracted mung bean protein + casein protein made into a chewable tablet	21.2								
Kohno et al. 2018 (pre study)	Intervention	6 Absence of disease (4M, 2F)		40.5 (9.1)			86.8 (14.7)	32.3 (4.8)			1.4 (0.4)					Mung bean protein tablet	Extracted mung bean protein + casein protein made into a chewable tablet	5.3								
	Intervention	8 Absence of disease (4M, 4F)		42.9 (8.2)			88.1 (14.4)	29.7 (10.1)			1.2 (0.3)					Mung bean protein tablet	Extracted mung bean protein + casein protein made into a chewable tablet	1.3								
	Control	8 Absence of disease (4M, 4F)		44.9 (13.6)			89.0 (13.3)	31.0 (6.2)			1.1 (0.5)					Milk protein tablet	Casein protein chewable tablet									

Study, Year	Intervention, Control	Participants number (M, F); health status	Gender info (Y/NR)	Mean Age, years (SD/Range)	Setting (IP/OP)	Country	BW (kg) (SD/range)	BMI (kg/m^2) (SD/range)	Mean LDL ± SD/range (mmol/L)	Mean HDL, mmol/L (SD/range)	Mean TG, mmol/L (SD/range)	Mean non-HDL, mmol/L (SD/range)	Mean ApoB, g/L (SD/range)	Design, washout length	Feeding Control[a]	Intervention or Comparator	Intervention and Food Source	Pulse protein dose (g/d)	Diet (% C:F:P)	SFA content (g/d; %E)	Fiber content (g/d; %E)	Energy Balance[b]	Energy Control[c]	Follow-up	Funding Sources[d]	Lipid Medication use (Y/N)
								Baseline																		
**Dried Peas**																										
Sirtori et al. 2011		136 Hypercholesterolaemic	NR		OP	Italy	NR						NR	P	Supp			34.6	54:24:17	9.1 (5.1)	33.8 (8.4)	Neutral	Substitution	4 wks	A	N
	Intervention	25 Hypercholesterolaemic		52.5 (12.7)				25.0 (2.1)	4.7 (0.7)	1.5 (0.4)	1.7 (0.9)	5.5 (0.8)				Pea protein + cellulose	Extracted protein incorporated into a snack bar									
	Intervention	23 Hypercholesterolaemic		55.3 (14.6)				25.6 (3.2)	5.2 (1.1)	1.5 (0.3)	1.8 (1.0)	6.0 (1.0)				Pea protein + oats										
	Intervention	21 Hypercholesterolaemic		53.9 (15.9)				25.3 (3.6)	4.5 (0.9)	1.5 (0.3)	1.6 (0.9)	5.3 (0.9)				Pea protein + pectin										
	Control	22 Hypercholesterolaemic		54.3 (12.8)				24.9 (3.4)	4.6 (0.8)	1.5 (0.4)	1.4 (0.5)	5.3 (0.9)				Milk protein + oats										
	Control	20 Hypercholesterolaemic		54.6 (15.5)				25.1 (3.0)	5.2 (1.5)	1.6 (0.3)	1.4 (0.5)	5.9 (1.3)				Milk protein + pectin										
Sucher et al. 2017			NR		OP	Germany	NR						NR	P	Supp & DA		Participants received daily food plans to ensure an equivalent intake of high amounts of protein	122.3	40:30:30		NR	Neutral	Substitution	6 wks	A	Unclear
	Intervention	19 Tye 2 Diabetes (12M, 7F)		63.7 (1.5)				29.4 (1.0)	3.4 (0.17)	1.09 (0.05)	1.64 (0.14)	4.15 (0.2)				High Plant Protien Diet (PP)				24.1 (9.3)						
	Control	18 Type 2 Diabetes (12M, 6F)		65.0 (1.4)				31 (0.8)	3.25 (0.22)	1.13 (0.07)	1.72 (0.13)	4.03 (0.3)				High Animal Protein Diet (AP)				27.6 (9.9)						
**Legume**																										
Crimarco et al. 2020		36 Absence of disease (12M, 24F)	NR	50.2 (13.8)	OP	USA	78.0 (17.6)	27.9 (5.2)	3.2 (0.9)	1.6 (0.3)	1.1 (0.4)	3.6 (1.0)	NR	C (no washout)	Supp & DA			34				Neutral	Substitution	8 wks	I	Unclear
	Intervention															Plant patties	All Plant products were supplied by Beyond Meat		42:41:19	26 (11.7)	27.9 (5.6)					
	Control															Animal patties	All Animal products were supplied by a San Francisco–based organic foods delivery service		40:44:18	33 (14.9)	22.3 (4.5)					

Abbreviations: A, agency; apoB, apolipoprotein B; BMI, body mass index; BW, body weight; C: F: P, carbohydrate: fat: protein; C, crossover; DA, dietary advice; F, female; HDL-C, high-density lipoprotein cholesterol; I, industry; LDL-C, low-density lipoprotein cholesterol; M, male; Met, metabolic feeding; NR, not reported; non-HDL-C, non-high-density lipoprotein cholesterol; OP, outpatient; P, parallel; Supp, supplemental feeding; TG, triglycerides; USA, United States of America.

[a] Supplemental feeding control (Supp) is the provision of some foods consumed during the study. Dietary Advice (DA) is the provision of advice or education on the food component of a specific diet.

[b] Neutral energy balance refers to the maintenance of usual energy intake. Positive energy balance refers to a greater than-normal energy intake. Negative energy balance refers to a deficit in normal energy intake.

[c] Energy control refers to the energy intake of the intervention group compared to the control group where substitution refers to energy matched between intervention and comparator, addition refers to excess energy between intervention and comparator, and subtraction refers to deficit in energy between intervention and comparator.

[d] Agency funding is that from government, university, or not-for-profit sources. Industry funding is that from trade organizations that obtain revenue from the sale of products.

Supplemental Table S5. Assessment of study product acceptability and adverse events*

Study	Assessment of Acceptability	Assessment of Adverse Events
Bahr et al. 2015	Participants reported similar palatability of study products (roll, bread, sausage, and spread) using lupin protein and milk protein (range: 1.7-2.6, average: 2.2, scale from best (1) to worst (6))	No adverse event observed
Sucher et al. 2017	NR	NR
Crimarco et al. 2020	Participants reported high satisfaction with both plant-based and animal-based meat (mean ≥ 3.5 with 5 being the highest)	No adverse event observed
Frota et al. 2015	Participants reported good acceptability of both the cowpea protein isolate and casein shakes	Participants reported flatulence (10.5%, n=4), obstipation (7.9%, n=3), and increase in stool softening (5.3%, n=2) after consuming cowpea protein; participants also reported flatulence (7.9 %, n = 3), obstipation (2.6 %, n = 1) and an increase in stool softening (5.3 %, n = 2) after consuming casein protein. Overall no drop out due to side effects
Sirtori et al. 2011	Participants dropped out from the study due to low satisfactory to the consumption of the protein bar (7.3%, n=14)	One participant dropped out due to minor gastrointestinal side effect
Weiße et al. 2010	Participants reported good acceptability of the lupin protein bars	NR
Kohno et al. 2018	NR	Participants reported dyspepsia (n=2), infrequent bowel movements (n=1), headache (n=1) and thirst (n=1) after consuming mung bean protein tablet; participants reported nausea (n=1), upper abdominal pain (n=1), and headache (n=1) in the control group

*Of the 7 trials, 5 reported some assessment of acceptability, and 5 reported some assessment of adverse events. Trials not listed in the table did not report acceptability and adverse events.
NR, not reported

Supplemental Table S6. Sensitivity analyses of the use of correlation coefficients of 0.25 and 0.75 for crossover trials in the primary analysis of the effect of extracted pulse proteins on blood lipids

	**MD (95% CI), P-value**<br>**I^2, P-value**		
	**Correlation Coefficient used in the Primary Analysis**	**Correlation Coefficient used in Sensitivity Analyses**	
**Outcomes (no. crossover trial comparisons/total)**	**0.5**	**0.25**	**0.75**
LDL-C (4/11)	-0.23 [-0.36, -0.10], P_{MD}<0.001<br>I^2=24.92%, P_Q=0.21	-0.24 [-0.37, 0.11], P_{MD}<0.001<br>I^2=19.3%, P_Q=0.26	-0.21 [-0.34, -0.08], P_{MD}=0.001<br>I^2=35.95%, P_Q=0.11
Non-HDL-C (4/11)	-0.22 [-0.36, -0.08], P_{MD}=0.002<br>I^2=54.21%, P_Q=0.02	-0.22 [-0.36, -0.08], P_{MD}=0.002<br>I^2=51.06%, P_Q=0.03	-0.21 [-0.35, -0.07], P_{MD}=0.002<br>I^2=60.51%, P_Q=0.00
ApoB (1/1)	-0.16 [-0.19, -0.13], P_{MD}<0.001	-0.16 [-0.19, -0.13], P_{MD}<0.001	-0.16 [-0.19, -0.13], P_{MD}<0.001
HDL-C (4/11)	0.03 [-0.00, 0.07], P_{MD}=0.076<br>I^2=0.00%, P_Q=0.91	0.03 [-0.00, 0.07], P_{MD}=0.072<br>I^2=0.00%, P_Q=0.93	0.03 [-0.01, 0.06], P_{MD}=0.156<br>I^2=0.00%, P_Q=0.86
TG (4/14)	-0.03 [-0.10, 0.05], P_{MD}=0.532<br>I^2=0.00%, P_Q=0.71	-0.02 [-0.10, 0.06], P_{MD}=0.555<br>I^2=0.00%, P_Q=0.71	-0.03 [-0.10, 0.05], P_{MD}=0.470<br>I^2=0.00%, P_Q=0.70

apoB, apolipoprotein B; CI, confidence interval; HDL-C, high-density lipoprotein cholesterol; LDL-C, low-density lipoprotein cholesterol; MD, mean difference; non-HDL-C, non-high-density lipoprotein cholesterol; TG, triglycerides.

Supplemental Table S7. GRADE assessment of the certainty of evidence

	GRADE assessment								
	Downgrades					Upgrades			
Outcome and trial (N)	ROB	Inconsistency	Indirectness	Imprecision	Publication bias	Dose response	Effect (MD [95% CI], P_{MD})	Certainty of Evidence[a]	Interpretation of magnitude of effect[b]
LDL-C (n=11)	Not serious	Not serious	Not serious	Not serious	None	None	↓ -0.23 mmol/L [-0.36 to -0.10], P<0.001	⊕⊕⊕⊕ HIGH	Moderate
Non-HDL-C (n=11)	Not serious	Not serious[1]	Not serious	Serious[2]	None	None	↓ -0.22 mmol/L [-0.36 to -0.08], P=0.002	⊕⊕⊕◯ MODERATE	Moderate
apoB (n=1)	Not serious	Not Serious	Very Serious[3]	Not serious	None[4]	None[5]	↓ -0.16 g/L [-0.19 to -0.13], P<0.001	⊕⊕◯◯ LOW	Trivial
HDL-C (n=11)	Not serious	Not serious	Not serious	Serious[6]	None	None	↔ 0.03 mmol/L [-0.00 to 0.07], P=0.076	⊕⊕⊕◯ MODERATE	No effect
TG (n=14)	Not serious	Not serious	Not serious	Serious[7]	None	None	↔ -0.03 mmol/L [-0.10 to 0.05], P=0.532	⊕⊕⊕◯ MODERATE	No effect

[a] Since all included trials were randomized controlled trials, the certainty of the evidence was graded as high for all outcomes by default and then downgraded or upgraded based on pre-specified criteria. Criteria for downgrades included risk of bias (downgraded if the majority of trials were considered to be at high risk of bias); inconsistency (downgraded if there was substantial unexplained heterogeneity [$I^2 \geq 50\%$, $p < 0.10$]; indirectness (downgraded if there were factors absent or present relating to the participants, interventions, or outcomes that limited the generalizability of the results); imprecision (downgraded if the 95% confidence interval crossed the minimally important difference [MID] for harm or benefit set as ± 0.1 mmol/L for LDL-C, non-HDL-C, HDL-C, and TG and ± 0.04 g/L for apoB, or there is a concern with robustness of the estimate resulting from sensitivity analyses); and publication bias (downgraded if there is evidence of publication bias based on funnel plot asymmetry and/or significant Egger's or Begg's tests (p<0.10) with confirmation by adjustment by Duval and Tweedie trim-and-fill analysis). Criteria for upgrades included a significant dose-response gradient.

[b] For the interpretation of the magnitude, we used the MIDs (see a above) to assess the importance of the magnitude of our pooled estimates using the effect size categories according to new GRADE guidance. We then used the MIDs to assess the importance of the magnitude of our point estimates using the effect size categories according to GRADE guidance as follows: large effect (≥5x MID); moderate effect (≥2x MID); small important effect (≥1x MID); and trivial/unimportant effect (<1 MID).

apoB, apolipoprotein B; CI, confidence interval; HDL-C, high-density lipoprotein cholesterol; LDL-C, low-density lipoprotein cholesterol; MD, mean difference; non-HDL-C, non-high-density lipoprotein cholesterol; ROB, risk of bias; TG, triglyceride.

[1] Although there was substantial heterogeneity in the analysis of the effect of extracted pulse proteins on non-HDL-C, we did not downgrade for serious inconsistency, since it was partially explained by the removal of Bähr et al. 2015 (milk protein) was removed (Original: I^2=54.21%, P_Q<0.02; after study removed: I^2=49%, P_Q=0.041), and Frota et al. 2015 (Original: I^2=54.21%, P_Q<0.02; after study removed: I^2=0%, P_Q=0.503).

[2] Downgrade assigned for imprecision. The 95% confidence interval is (-0.36 to -0.08), which crossed the MID for benefit (-0.1mmol/L).

[3] Double downgrade for serious indirectness as the one trial comparison comes from one study of hypercholesterolemic adults (mean 57y), which leads to poor generalizability of the results to the adult population.

[4] No downgrade for publication bias, as publication bias could not be assessed due to lack of power for assessing funnel plot asymmetry and small study effects (<10 trial comparisons included in the meta-analysis).

[5] No upgrade for dose-response, as dose-response could not be assessed as <6 trials were available.

[6] Downgrade for serious imprecision due to gain of significance in sensitivity analyses with the removal of Bähr et al. 2015 (recalculated MD: 0.04; 95% CI: -0.00, 0.08; P_{MD}=0.045)

[7] Downgrade assigned for imprecision. The 95% confidence interval is (-0.104 to 0.054), which crossed the MID for benefit (-0.1mmol/L).

Supplemental Figure S1. Risk of bias proportion graph for the effect of extracted pulse proteins on blood lipids in parallel trials

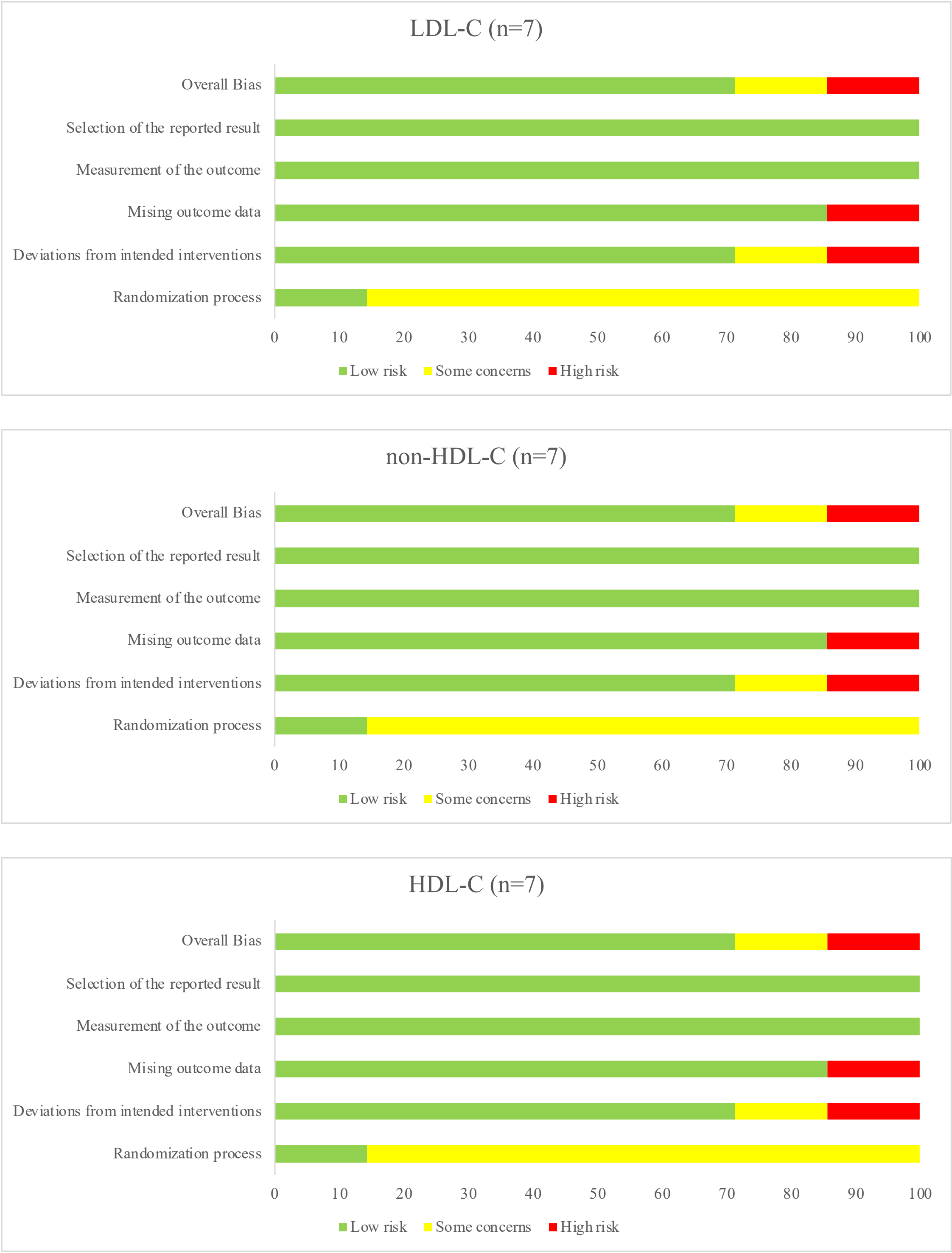

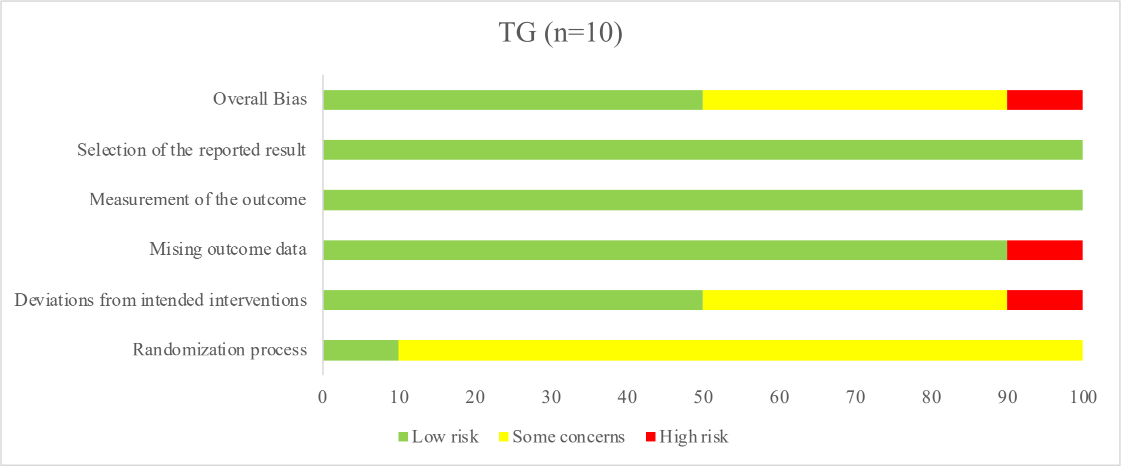

apoB, apolipoprotein B; HDL-C, high-density lipoprotein cholesterol; LDL-C, low-density lipoprotein cholesterol; non-HDL-C, non-high-density lipoprotein cholesterol; TG, triglyceride.

Supplemental Figure S2. Risk of bias proportion graph for the effect of extracted pulse proteins on blood lipids in crossover trials

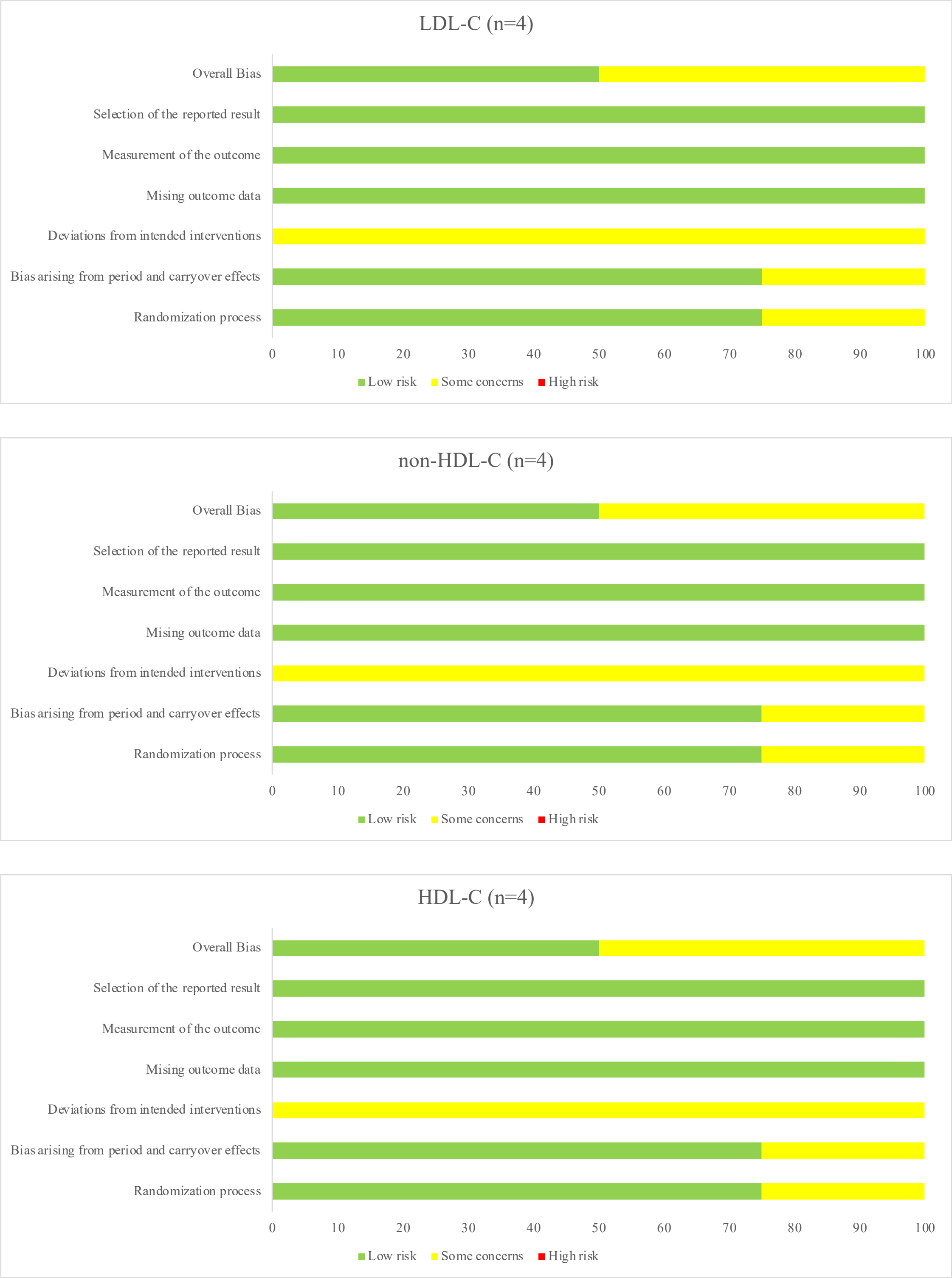

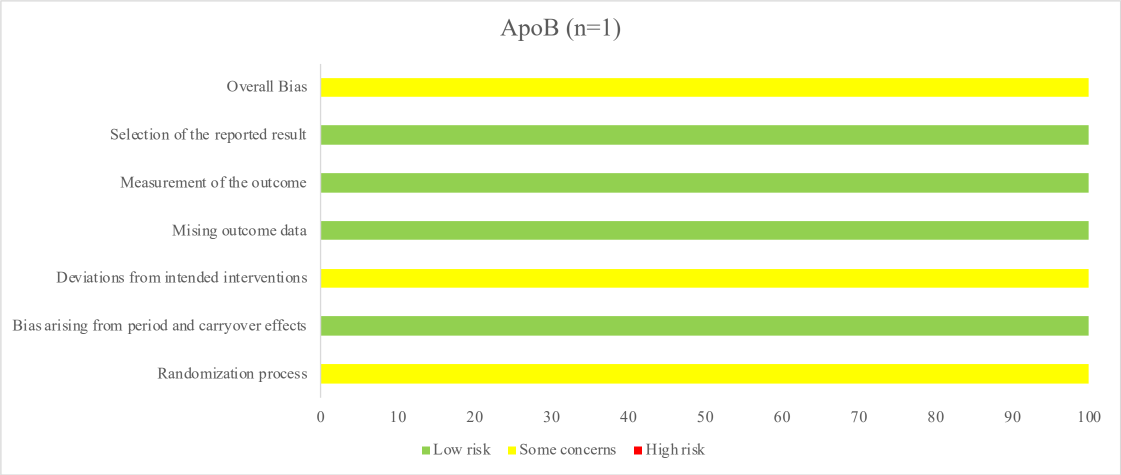

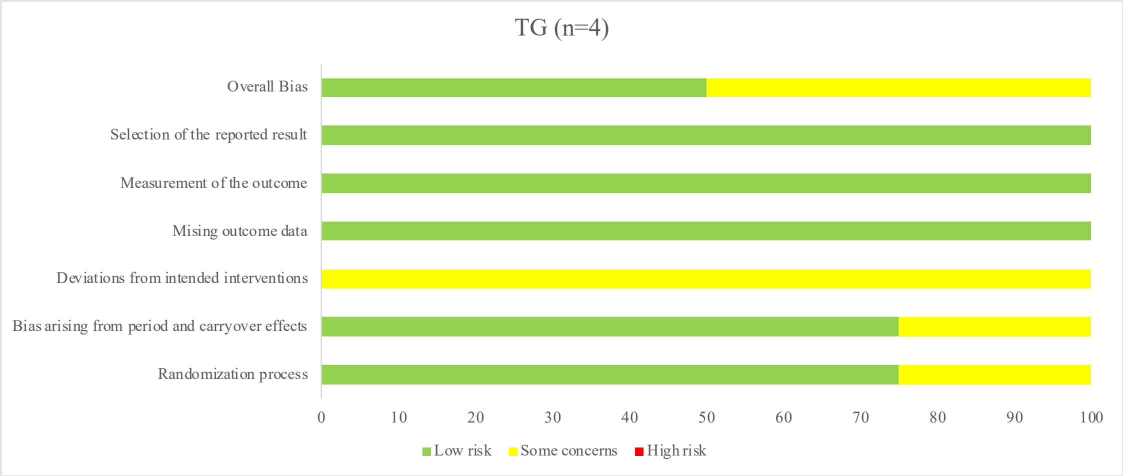

apoB, apolipoprotein B; HDL-C, high-density lipoprotein cholesterol; LDL-C, low-density lipoprotein cholesterol; non-HDL-C, non-high-density lipoprotein cholesterol; TG, triglyceride.

Supplemental Figure S3. Forest plot of randomized controlled trials of the effect of extracted pulse proteins on LDL-C

LDL-C (mmol/L)

Trial or subgroup	N Control	N Intervention	Mean difference with 95% CI	Weight (%)	A	B	C	D	E	F	G
**Beans**											
Weiße et al. 2010	21	22	-0.16 [-0.52 to 0.20]	9.70	S		H	H	L	L	H
Sirtori et al. 2011 (lupin+cellulose)	25	22	-0.27 [-0.90 to 0.35]	3.78	S		L	L	L	L	L
Bähr et al. 2015 (milk protein with arg)	68	68	0.01 [-0.31 to 0.33]	11.19	L	L	S	L	L	L	L
Bähr et al. 2015 (milk protein)	68	68	-0.17 [-0.49 to 0.15]	11.51	L	L	S	L	L	L	L
Frota et al. 2015	38	38	-0.61 [-0.88 to -0.33]	14.04	S	L	S	L	L	L	S
Kohno et al. 2018 (main study)	22	22	-0.21 [-0.63 to 0.21]	7.49	S		S	L	L	L	S
Heterogeneity: $\tau^2 = 0.03$, $I^2 = 47.80\%$, $H^2 = 1.92$			-0.24 [-0.45 to -0.04]								
Test of $\theta_i = \theta_j$: Q(5) = 9.58, p = 0.09											
**Dried peas**											
Sirtori et al. 2011 (pea protein+cellulose)	25	25	0.09 [-0.48 to 0.66]	4.40	S		L	L	L	L	L
Sirtori et al. 2011 (pea protein+oat fibre)	22	23	-0.37 [-0.89 to 0.14]	5.31	S		L	L	L	L	L
Sirtori et al. 2011 (pea protein+pectin)	20	21	-0.18 [-0.82 to 0.45]	3.65	S		L	L	L	L	L
Sucher et al. 2017	18	19	0.14 [-0.40 to 0.68]	4.86	L		L	L	L	L	L
Heterogeneity: $\tau^2 = 0.00$, $I^2 = 0.00\%$, $H^2 = 1.00$			-0.09 [-0.37 to 0.19]								
Test of $\theta_i = \theta_j$: Q(3) = 2.30, p = 0.51											
**Legume intervention**											
Crimarco et al. 2020	36	36	-0.28 [-0.44 to -0.12]	24.09	L	S	S	L	L	L	S
Heterogeneity: $\tau^2 = 0.00$, $I^2 = .\%$, $H^2 = .$			-0.28 [-0.44 to -0.12]								
Test of $\theta_i = \theta_j$: Q(0) = 0.00, p = .											
**Overall**			-0.23 [-0.36 to -0.10]								
Heterogeneity: $\tau^2 = 0.01$, $I^2 = 24.92\%$, $H^2 = 1.33$											
Test of $\theta_i = \theta_j$: Q(10) = 13.32, p = 0.21											
Test of group differences: p =0.636, $I^2 = 32.66$, p_Q =0.157											

(Risk of bias columns A–G)

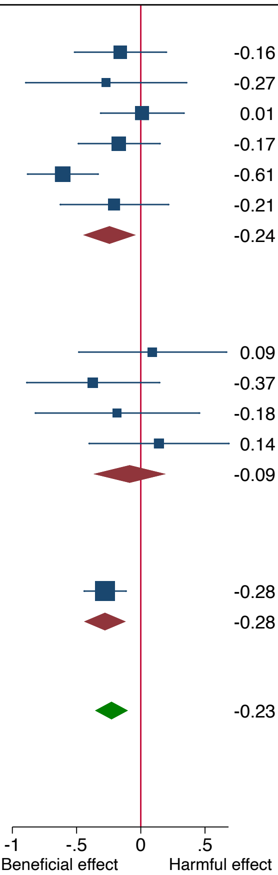

Test of $\theta = 0$: z = -3.497, p < 0.001

Pooled effect estimates for each subgroup and overall effect are represented by the diamonds. Data are expressed as weighted mean differences with 95% confidence intervals using the generic inverse-variance method and random effects DerSimonian-Laird model. Paired analyses were applied to all crossover trials. Inter-study heterogeneity was assessed using the Cochran Q statistic and quantified using the I^2 statistic, with significance set at p<0.100 and $I^2 \geq 50\%$ considered to be evidence of substantial heterogeneity.

Risk of Bias Legend: (H) High Risk; (L) Low Risk; (S) Some concerns. The letters represent the following risk of bias domains: A, randomization process; B, bias arising from period and carryover effects; C, deviations from intended intervention; D, missing outcome data; E, measurement of the outcome; F, selection of the reported result; and G, overall bias. Risk of bias arising from period and carryover effects was only applicable to crossover trials.

The pooled effect summary was calculated with the $\chi 2$ test. The test for group differences was calculated with meta-regression, which uses the Wald test.

CI, confidence interval; LDL-C, Low-density lipoprotein cholesterol

Supplemental Figure S4. Forest plot of randomized controlled trials of the effect of extracted pulse proteins on non-HDL-C

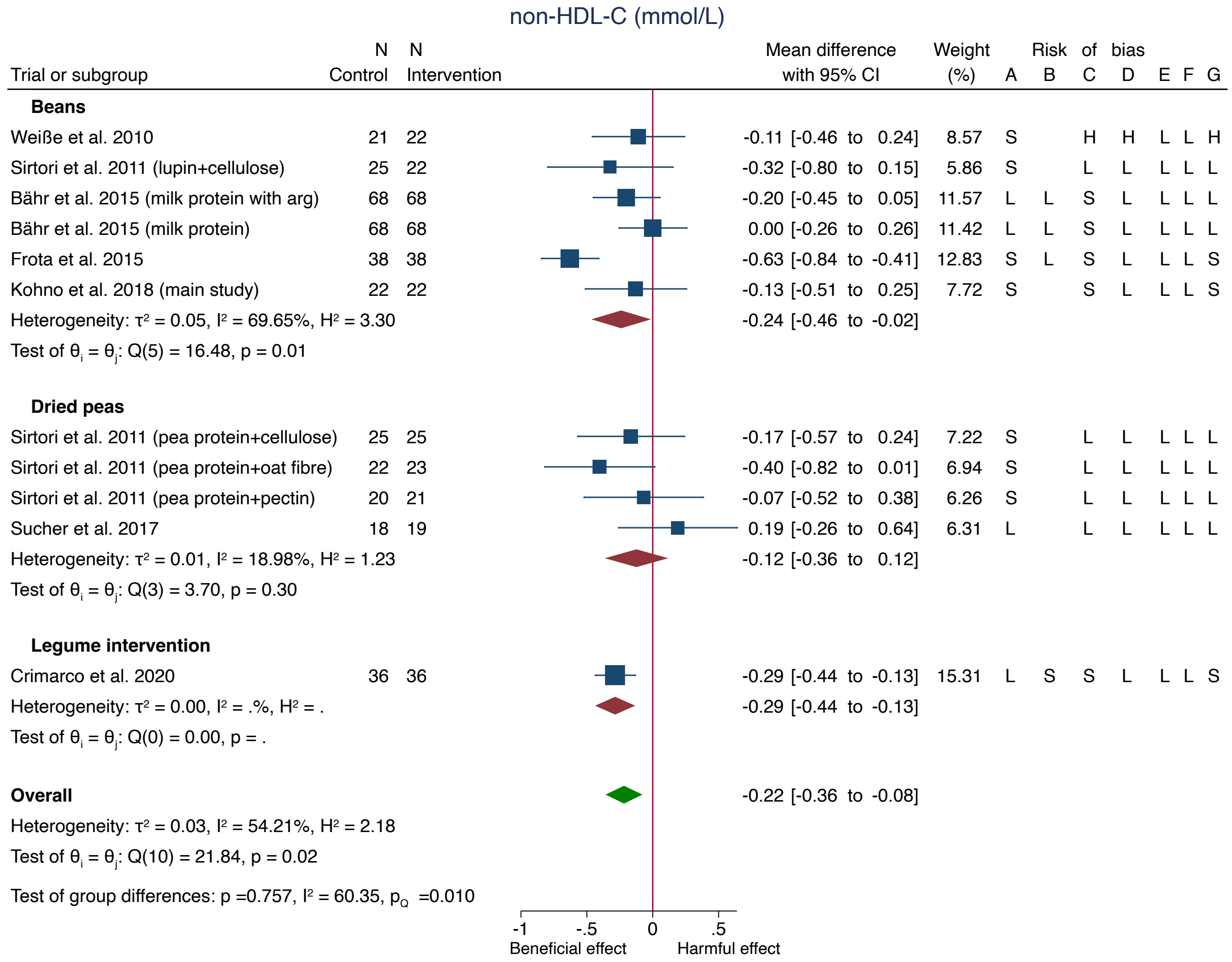

Test of θ = 0: z = -3.084, p =0.002

Pooled effect estimates for each subgroup and overall effect are represented by the diamonds. Data are expressed as weighted mean differences with 95% confidence intervals using the generic inverse-variance method and random effects DerSimonian-Laird model. Paired analyses were applied to all crossover trials. Inter-study heterogeneity was assessed using the Cochran Q statistic and quantified using the I^2 statistic, with significance set at p<0.100 and $I^2 \geq 50\%$ considered to be evidence of substantial heterogeneity.

Risk of Bias Legend: (H) High Risk; (L) Low Risk; (S) Some concerns. The letters represent the following risk of bias domains: A, randomization process; B, bias arising from period and carryover effects; C, deviations from intended intervention; D, missing outcome data; E, measurement of the outcome; F, selection of the reported result; and G, overall bias. Risk of bias arising from period and carryover effects was only applicable to crossover trials.

The pooled effect summary was calculated with the χ2 test. The test for group differences was calculated with meta-regression, which uses the Wald test.

CI, confidence interval; non-HDL-C, non-high density lipoprotein cholesterol

Supplemental Figure S5. Forest plot of randomized controlled trials of the effect of extracted pulse proteins on HDL-C

HDL-C(mmol/L)

Trial or subgroup	N Control	N Intervention	Mean difference with 95% CI	Weight (%)	Risk of bias A	B	C	D	E	F	G
**Beans**											
Weiße et al. 2010	21	22	0.09 [-0.03 to 0.21]	8.91	S		H	H	L	L	H
Sirtori et al. 2011 (lupin+cellulose)	25	22	-0.05 [-0.29 to 0.19]	2.20	S		L	L	L	L	L
Bähr et al. 2015 (milk protein with arg)	68	68	-0.03 [-0.15 to 0.09]	8.40	L	L	S	L	L	L	L
Bähr et al. 2015 (milk protein)	68	68	0.05 [-0.07 to 0.17]	8.27	L	L	S	L	L	L	L
Frota et al. 2015	38	38	0.08 [0.00 to 0.16]	20.61	S	L	S	L	L	L	S
Kohno et al. 2018 (main study)	22	22	-0.00 [-0.09 to 0.09]	15.93	S		S	L	L	L	S
Heterogeneity: $\tau^2 = 0.00$, $I^2 = 0.00\%$, $H^2 = 1.00$			0.04 [-0.00 to 0.08]								
Test of $\theta_i = \theta_j$: Q(5) = 4.38, p = 0.50											
**Dried peas**											
Sirtori et al. 2011 (pea protein+cellulose)	25	25	0.02 [-0.21 to 0.26]	2.28	S		L	L	L	L	L
Sirtori et al. 2011 (pea protein+oat fibre)	22	23	0.01 [-0.20 to 0.22]	2.89	S		L	L	L	L	L
Sirtori et al. 2011 (pea protein+pectin)	20	21	0.03 [-0.17 to 0.23]	3.18	S		L	L	L	L	L
Sucher et al. 2017	18	19	0.02 [-0.13 to 0.17]	5.81	L		L	L	L	L	L
Heterogeneity: $\tau^2 = 0.00$, $I^2 = 0.00\%$, $H^2 = 1.00$			0.02 [-0.08 to 0.12]								
Test of $\theta_i = \theta_j$: Q(3) = 0.02, p = 1.00											
**Legume intervention**											
Crimarco et al. 2020	36	36	0.02 [-0.06 to 0.10]	21.51	L	S	S	L	L	L	S
Heterogeneity: $\tau^2 = 0.00$, $I^2 = .\%$, $H^2 = .$			0.02 [-0.06 to 0.10]								
Test of $\theta_i = \theta_j$: Q(0) = -0.00, p = .											
**Overall**			0.03 [-0.00 to 0.07]								
Heterogeneity: $\tau^2 = 0.00$, $I^2 = 0.00\%$, $H^2 = 1.00$											
Test of $\theta_i = \theta_j$: Q(10) = 4.71, p = 0.91											
Test of group differences: p =0.856, $I^2 = 0.00$, p_Q =0.820											

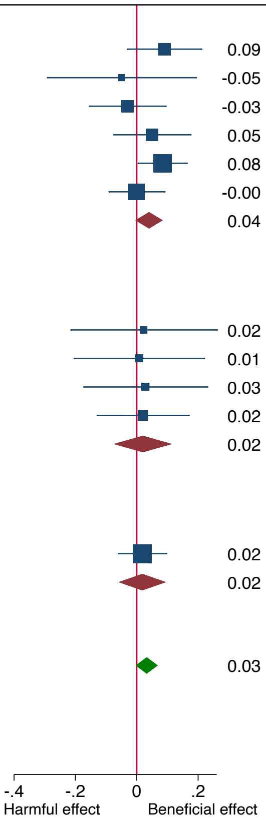

Test of θ = 0: z = 1.776, p =0.076

Pooled effect estimates for each subgroup and overall effect are represented by the diamonds. Data are expressed as weighted mean differences with 95% confidence intervals using the generic inverse-variance method and random effects DerSimonian-Laird model. Paired analyses were applied to all crossover trials. Inter-study heterogeneity was assessed using the Cochran Q statistic and quantified using the I^2 statistic, with significance set at p<0.100 and $I^2 \geq 50\%$ considered to be evidence of substantial heterogeneity.

Risk of Bias Legend: (H) High Risk; (L) Low Risk; (S) Some concerns. The letters represent the following risk of bias domains: A, randomization process; B, bias arising from period and carryover effects; C, deviations from intended intervention; D, missing outcome data; E, measurement of the outcome; F, selection of the reported result; and G, overall bias. Risk of bias arising from period and carryover effects was only applicable to crossover trials.

The pooled effect summary was calculated with the $\chi 2$ test. The test for group differences was calculated with meta-regression, which uses the Wald test.

CI, confidence interval; HDL-C, high density lipoprotein cholesterol

Supplemental Figure S6. Forest plot of randomized controlled trials of the effect of extracted pulse proteins on TG

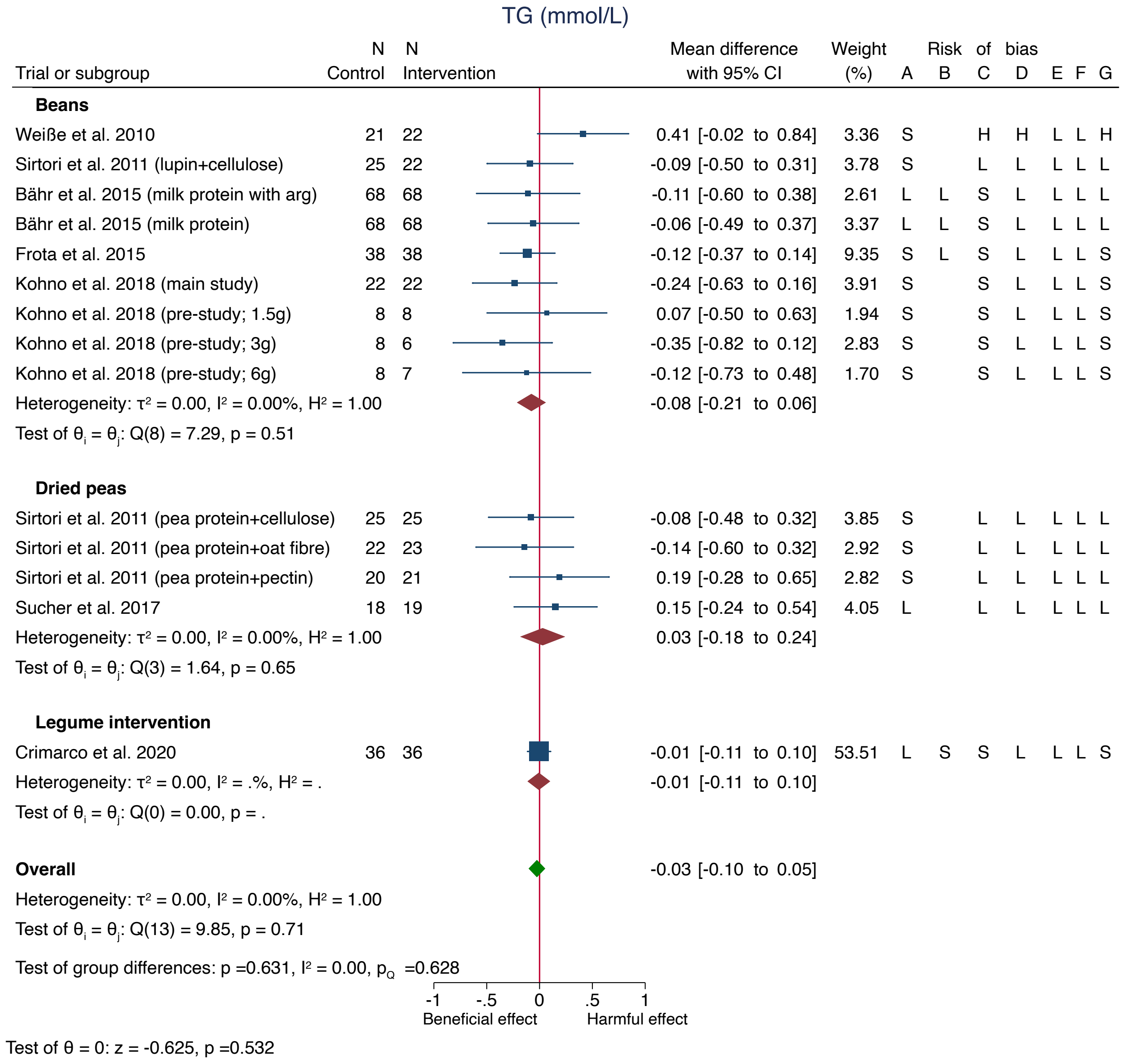

Pooled effect estimates for each subgroup and overall effect are represented by the diamonds. Data are expressed as weighted mean differences with 95% confidence intervals using the generic inverse-variance method and random effects DerSimonian-Laird model. Paired analyses were applied to all crossover trials. Inter-study heterogeneity was assessed using the Cochran Q statistic and quantified using the I^2 statistic, with significance set at $p<0.100$ and $I^2 \geq 50\%$ considered to be evidence of substantial heterogeneity.

Risk of Bias Legend: (H) High Risk; (L) Low Risk; (S) Some concerns. The letters represent the following risk of bias domains: A, randomization process; B, bias arising from period and carryover effects; C, deviations from intended intervention; D, missing outcome data; E, measurement of the outcome; F, selection of the reported result; and G, overall bias. Risk of bias arising from period and carryover effects was only applicable to crossover trials.

The pooled effect summary was calculated with the $\chi 2$ test. The test for group differences was calculated with meta-regression, which uses the Wald test.

CI, confidence interval; TG, triglyceride

Supplemental Figure S7. Sensitivity analysis of the systematic removal of each trial for the effect of extracted pulse proteins on LDL-C

Influence analysis

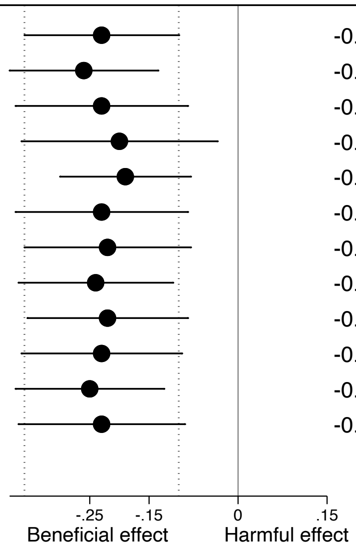

Trial removed	Mean difference with 95% CI	P_{MD}	I^2 (%)	P_Q
**Overall**	-0.23 [-0.36 to -0.10]	< 0.001	25	0.206
Bähr et al. 2015 (milk protein with arg)	-0.26 [-0.38 to -0.13]	< 0.001	15	0.301
Bähr et al. 2015 (milk protein)	-0.23 [-0.38 to -0.09]	0.002	31	0.159
Crimarco et al. 2020	-0.20 [-0.37 to -0.04]	0.012	31	0.160
Frota et al. 2015	-0.19 [-0.30 to -0.08]	< 0.001	0	0.783
Kohno et al. 2018 (main study)	-0.23 [-0.38 to -0.09]	0.002	32	0.150
Sirtori et al. 2011 (lupin+cellulose)	-0.22 [-0.36 to -0.08]	0.002	32	0.149
Sirtori et al. 2011 (pea protein+cellulose)	-0.24 [-0.37 to -0.11]	< 0.001	25	0.216
Sirtori et al. 2011 (pea protein+oat fibre)	-0.22 [-0.35 to -0.09]	0.002	31	0.159
Sirtori et al. 2011 (pea protein+pectin)	-0.23 [-0.37 to -0.09]	0.001	32	0.150
Sucher et al. 2017	-0.25 [-0.38 to -0.12]	< 0.001	20	0.256
Weiße et al. 2010	-0.23 [-0.37 to -0.09]	0.002	31	0.159

Influence analysis: removal of each trial, one at a time and recalculation of the overall effect and heterogeneity

arg, arginine; CI, confidence interval; LDL-C, low-density lipoprotein cholesterol

Supplemental Figure S8. Sensitivity analysis of the systematic removal of each trial for the effect of extracted pulse proteins on non-HDL-C

Influence analysis

Trial removed	Mean difference with 95% CI	P_{MD}	I^2 (%)	P_Q
**Overall**	-0.22 [-0.36 to -0.08]	0.002	54	0.016
Bähr et al. 2015 (milk protein with arg)	-0.22 [-0.38 to -0.06]	0.007	58	0.010
Bähr et al. 2015 (milk protein)	-0.25 [-0.39 to -0.11]	< 0.001	49	0.041
Crimarco et al. 2020	-0.20 [-0.37 to -0.03]	0.019	58	0.010
Frota et al. 2015	-0.19 [-0.28 to -0.09]	< 0.001	0	0.503
Kohno et al. 2018 (main study)	-0.22 [-0.37 to -0.07]	0.003	58	0.011
Sirtori et al. 2011 (lupin+cellulose)	-0.21 [-0.36 to -0.06]	0.005	59	0.010
Sirtori et al. 2011 (pea protein+cellulose)	-0.22 [-0.37 to -0.07]	0.004	58	0.010
Sirtori et al. 2011 (pea protein+oat fibre)	-0.20 [-0.35 to -0.05]	0.007	58	0.011
Sirtori et al. 2011 (pea protein+pectin)	-0.23 [-0.38 to -0.09]	0.002	57	0.012
Sucher et al. 2017	-0.25 [-0.38 to -0.12]	< 0.001	50	0.036
Weiße et al. 2010	-0.23 [-0.38 to -0.08]	0.003	57	0.012

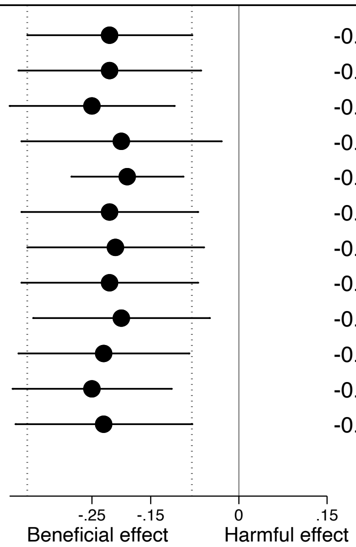

Influence analysis: removal of each trial, one at a time and recalculation of the overall effect and heterogeneity

arg, arginine; CI, confidence interval; non-HDL-C, non-high density lipoprotein cholesterol

Supplemental Figure S9. Sensitivity analysis of the systematic removal of each trial for the effect of extracted pulse proteins on HDL-C

Influence analysis

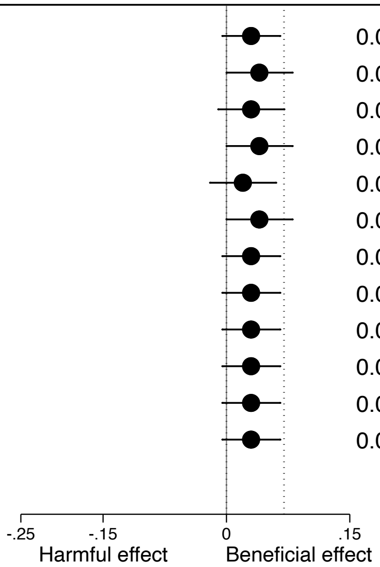

Trial removed	Mean difference with 95% CI	P_{MD}	I^2 (%)	P_Q
**Overall**	0.03 [-0.01 to 0.06]	0.076	0	0.910
Bähr et al. 2015 (milk protein with arg)	0.04 [-0.00 to 0.08]	0.045	0	0.934
Bähr et al. 2015 (milk protein)	0.03 [-0.01 to 0.07]	0.105	0	0.866
Crimarco et al. 2020	0.04 [-0.00 to 0.08]	0.078	0	0.873
Frota et al. 2015	0.02 [-0.02 to 0.06]	0.349	0	0.976
Kohno et al. 2018 (main study)	0.04 [-0.00 to 0.08]	0.053	0	0.904
Sirtori et al. 2011 (lupin+cellulose)	0.03 [-0.01 to 0.06]	0.063	0	0.893
Sirtori et al. 2011 (pea protein+cellulose)	0.03 [-0.01 to 0.06]	0.077	0	0.859
Sirtori et al. 2011 (pea protein+oat fibre)	0.03 [-0.01 to 0.06]	0.073	0	0.863
Sirtori et al. 2011 (pea protein+pectin)	0.03 [-0.01 to 0.06]	0.079	0	0.859
Sucher et al. 2017	0.03 [-0.01 to 0.06]	0.078	0	0.861
Weiße et al. 2010	0.03 [-0.00 to 0.06]	0.161	0	0.927

Influence analysis: removal of each trial, one at a time and recalculation of the overall effect and heterogeneity

arg, arginine; CI, confidence interval; HDL-C, high-density lipoprotein cholesterol

Supplemental Figure S10. Sensitivity analysis of the systematic removal of each trial for the effect of extracted pulse proteins on TG

Influence analysis

Trial removed	Mean difference with 95% CI	P_{MD}	I^2 (%)	P_Q
**Overall**	-0.03 [-0.11 to 0.05]	0.532	0	0.706
Bähr et al. 2015 (milk protein with arg)	-0.02 [-0.10 to 0.06]	0.575	0	0.639
Bähr et al. 2015 (milk protein)	-0.02 [-0.10 to 0.06]	0.559	0	0.631
Crimarco et al. 2020	-0.05 [-0.16 to 0.06]	0.433	0	0.650
Frota et al. 2015	-0.02 [-0.11 to 0.07]	0.709	0	0.675
Kohno et al. 2018 (main study)	-0.02 [-0.10 to 0.06]	0.688	0	0.728
Kohno et al. 2018 (pre-study; 1.5g)	-0.03 [-0.11 to 0.05]	0.507	0	0.638
Kohno et al. 2018 (pre-study; 3g)	-0.02 [-0.10 to 0.06]	0.702	0	0.790
Kohno et al. 2018 (pre-study; 6g)	-0.02 [-0.10 to 0.06]	0.564	0	0.638
Sirtori et al. 2011 (lupin+cellulose)	-0.02 [-0.10 to 0.06]	0.584	0	0.639
Sirtori et al. 2011 (pea protein+cellulose)	-0.02 [-0.10 to 0.06]	0.578	0	0.636
Sirtori et al. 2011 (pea protein+oat fibre)	-0.02 [-0.10 to 0.06]	0.597	0	0.652
Sirtori et al. 2011 (pea protein+pectin)	-0.03 [-0.11 to 0.05]	0.443	0	0.699
Sucher et al. 2017	-0.03 [-0.11 to 0.05]	0.428	0	0.699
Weiße et al. 2010	-0.04 [-0.12 to 0.04]	0.325	0	0.928

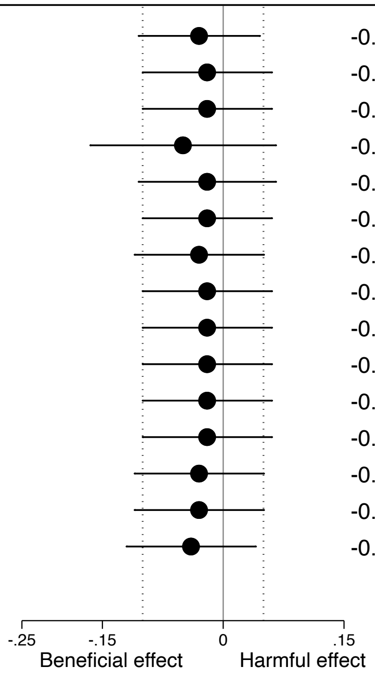

Influence analysis: removal of each trial, one at a time and recalculation of the overall effect and heterogeneity

arg, arginine; CI, confidence interval; TG, triglyceride

Supplemental Figure S11 (1 of 3). Subgroup analyses for the effect of extracted pulse proteins on LDL-C

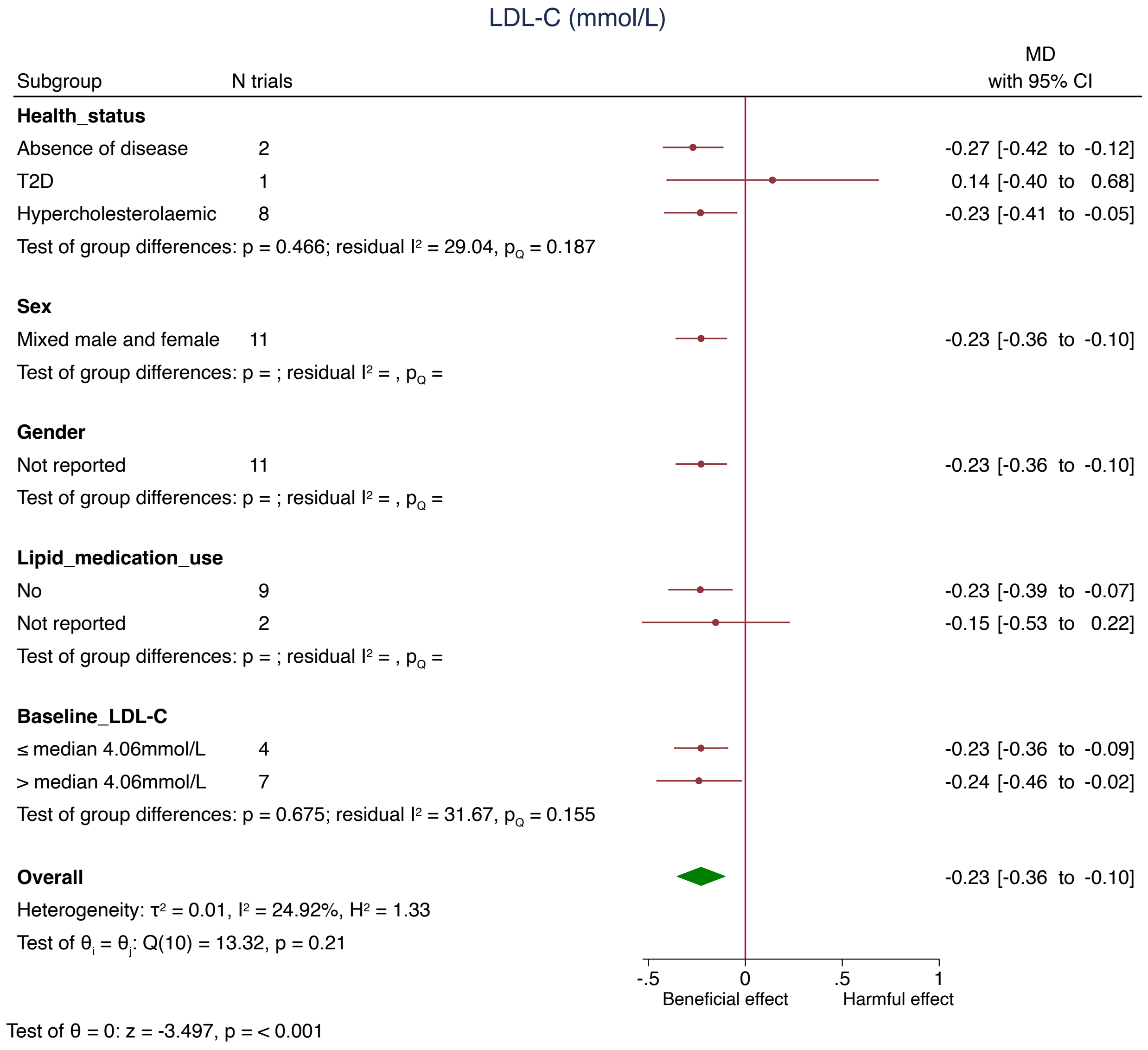

Pooled effect estimates for each subgroup are represented as red circles and the overall effect estimate as the green diamond. Data are expressed as weighted mean differences with 95% confidence intervals using the generic inverse-variance method and random effects DerSimonian-Laird model. Paired analyses were applied to all crossover trials. Inter-study heterogeneity was assessed using the Cochran Q statistic and quantified using the I^2 statistic, with significance set at p<0.100 and $I^2 \geq 50\%$ considered to be evidence of substantial heterogeneity.
The pooled effect summary was calculated with the χ^2 test. The test for subgroup differences was calculated with meta-regression, which uses the Wald test.

CI, confidence interval; LDL-C, low-density cholesterol; MD, mean difference; T2D, type 2 diabetes

Supplemental Figure S11 (2 of 3). Subgroup analyses for the effect of extracted pulse proteins on LDL-C

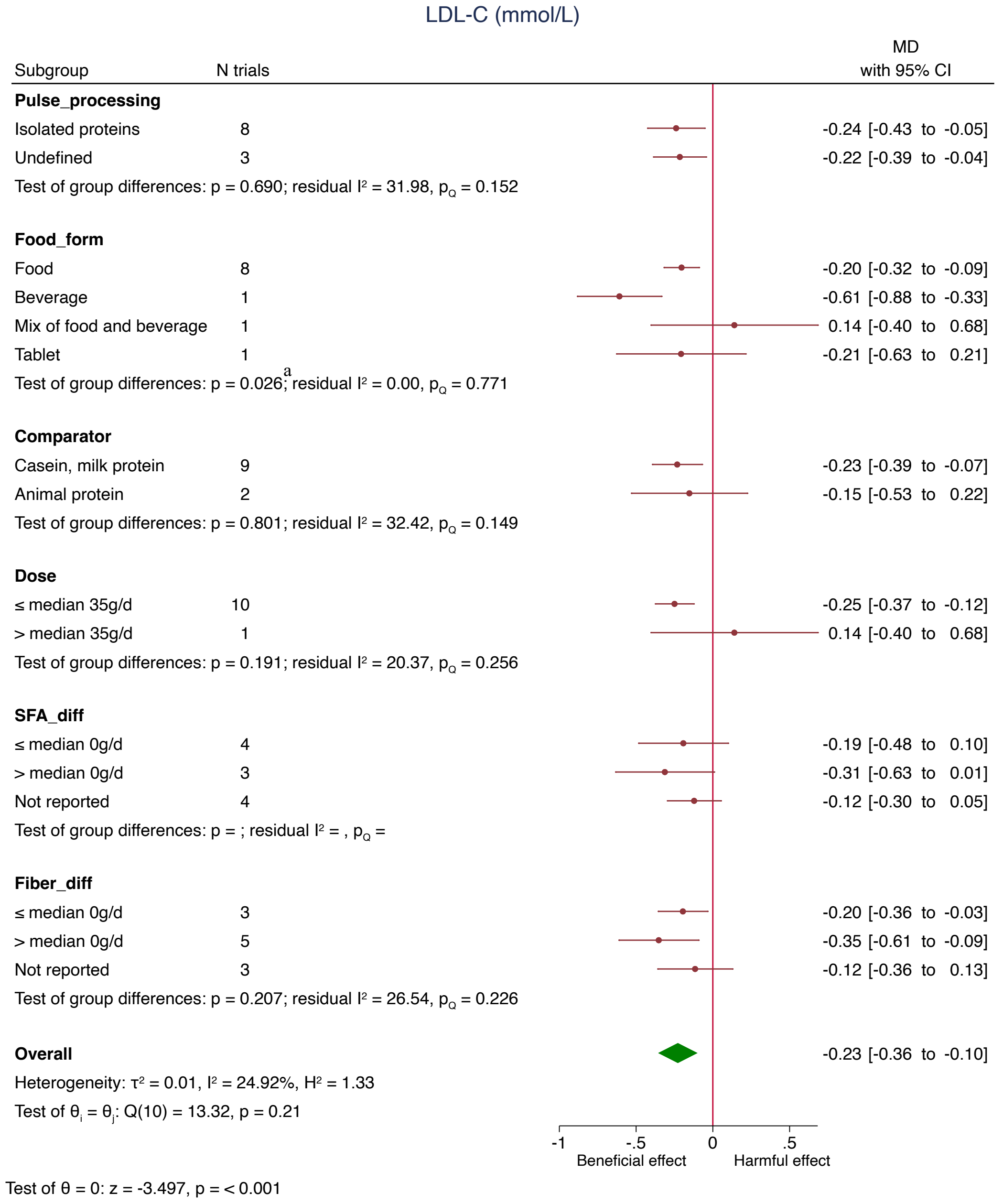

Pooled effect estimates for each subgroup are represented as red circles and the overall effect estimate as the green diamond. Data are expressed as weighted mean differences with 95% confidence intervals using the generic inverse-variance method and random effects DerSimonian-Laird model. Paired analyses were applied to all crossover trials. Inter-study heterogeneity was assessed using the Cochran Q statistic and quantified using the I^2 statistic, with significance set at $p<0.100$ and $I^2 \geq 50\%$ considered to be evidence of substantial heterogeneity.
The pooled effect summary was calculated with the χ^2 test. The test for subgroup differences was calculated with meta-regression, which uses the Wald test.
SFA_diff refers to the difference in SFA (g/d) of the overall diet between the intervention and control; Fiber_diff refers to the difference in fiber (g/d) of the overall diet between the intervention and control
[a] Pairwise between-subgroup mean differences (95% confidence intervals) for Food form were as follows: Food vs. Beverage -0.405 mmol/L (-0.701, -0.108); Food vs, Mix of food and beverage 0.344 mmol/L (-0.211, 0.899); Food vs. Tablet -0.00302 mmol/L (-0.439, 0.433); Beverage vs. Mix of food and beverage 0.748 mmol/L (0.14, 1.36); Beverage vs. Tablet 0.402 mmol/L (-0.1, 0.903); Miz of food and beverage vs. Tablet 0.347 mmol/L (-1.03, 0.34)

CI, confidence interval; LDL-C, low-density cholesterol; MD, mean difference

Supplemental Figure S11 (3 of 3). Subgroup analyses for the effect of extracted pulse proteins on LDL-C

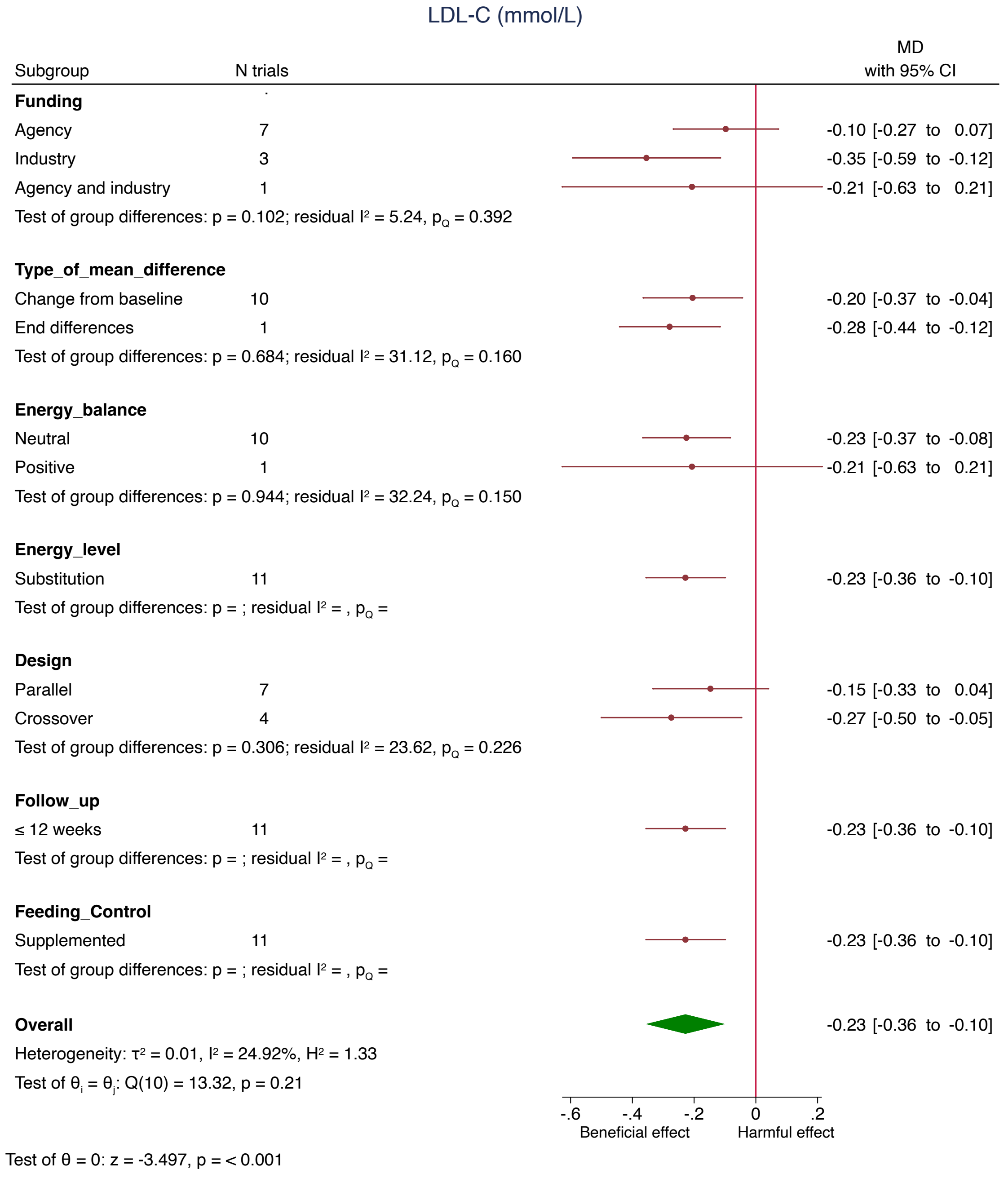

Pooled effect estimates for each subgroup are represented as red circles and the overall effect estimate as the green diamond. Data are expressed as weighted mean differences with 95% confidence intervals using the generic inverse-variance method and random effects DerSimonian-Laird model. Paired analyses were applied to all crossover trials. Inter-study heterogeneity was assessed using the Cochran Q statistic and quantified using the I^2 statistic, with significance set at $p<0.100$ and $I^2 \geq 50\%$ considered to be evidence of substantial heterogeneity.
The pooled effect summary was calculated with the χ^2 test. The test for subgroup differences was calculated with meta-regression, which uses the Wald test.

CI, confidence interval; LDL-C, low-density cholesterol; MD, mean difference

Supplemental Figure S12 (1 of 3). Subgroup analyses for the effect of extracted pulse proteins on non-HDL-C

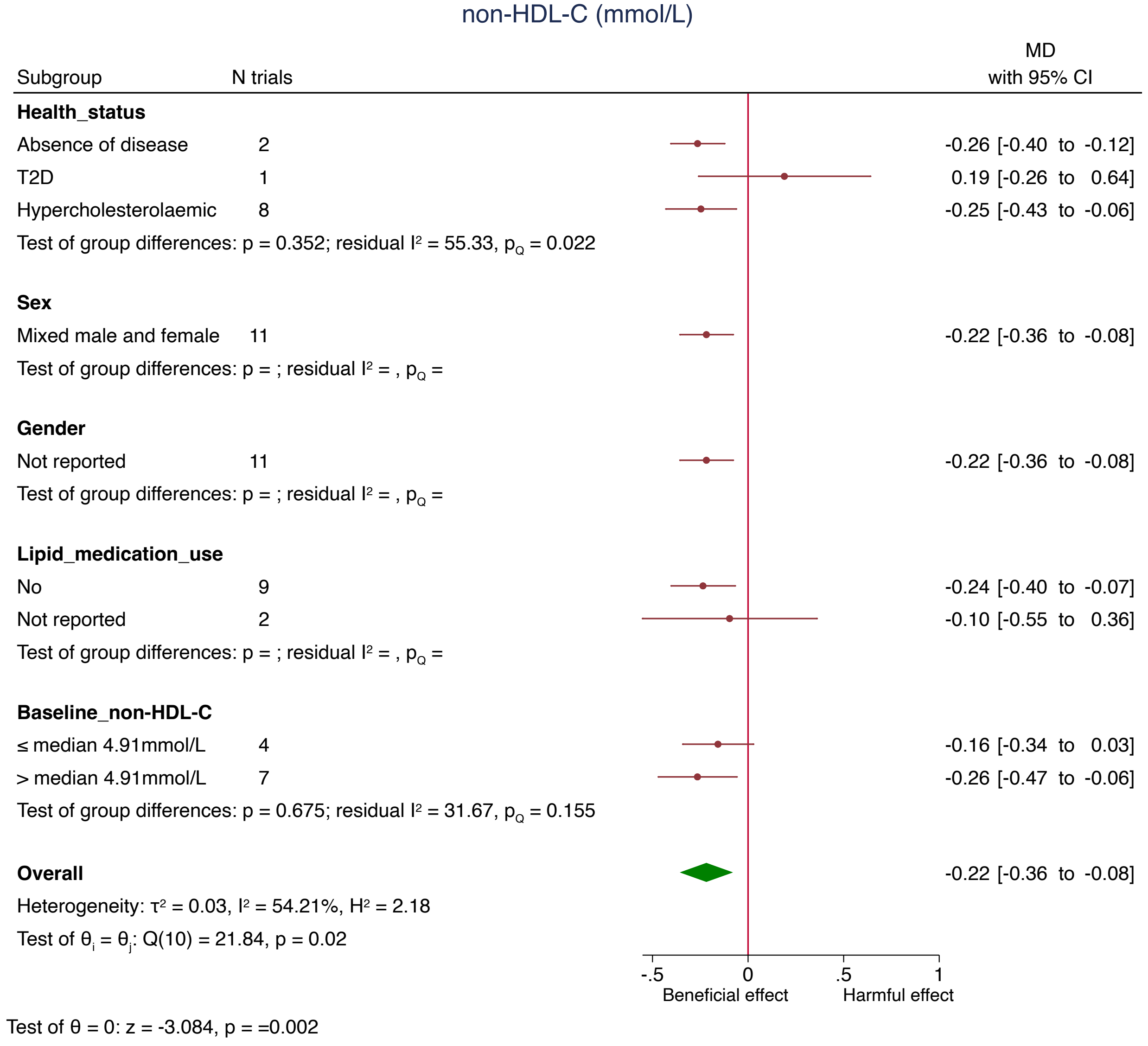

Pooled effect estimates for each subgroup are represented as red circles and the overall effect estimate as the green diamond. Data are expressed as weighted mean differences with 95% confidence intervals using the generic inverse-variance method and random effects DerSimonian-Laird model. Paired analyses were applied to all crossover trials. Inter-study heterogeneity was assessed using the Cochran Q statistic and quantified using the I^2 statistic, with significance set at $p<0.100$ and $I^2 \geq 50\%$ considered to be evidence of substantial heterogeneity.
The pooled effect summary was calculated with the χ^2 test. The test for subgroup differences was calculated with meta-regression, which uses the Wald test.

CI, confidence interval; MD, mean difference; non-HDL-C, non-high density lipoprotein cholesterol; T2D, type 2 diabetes

Supplemental Figure S12 (2 of 3). Subgroup analyses for the effect of extracted pulse proteins on non-HDL-C

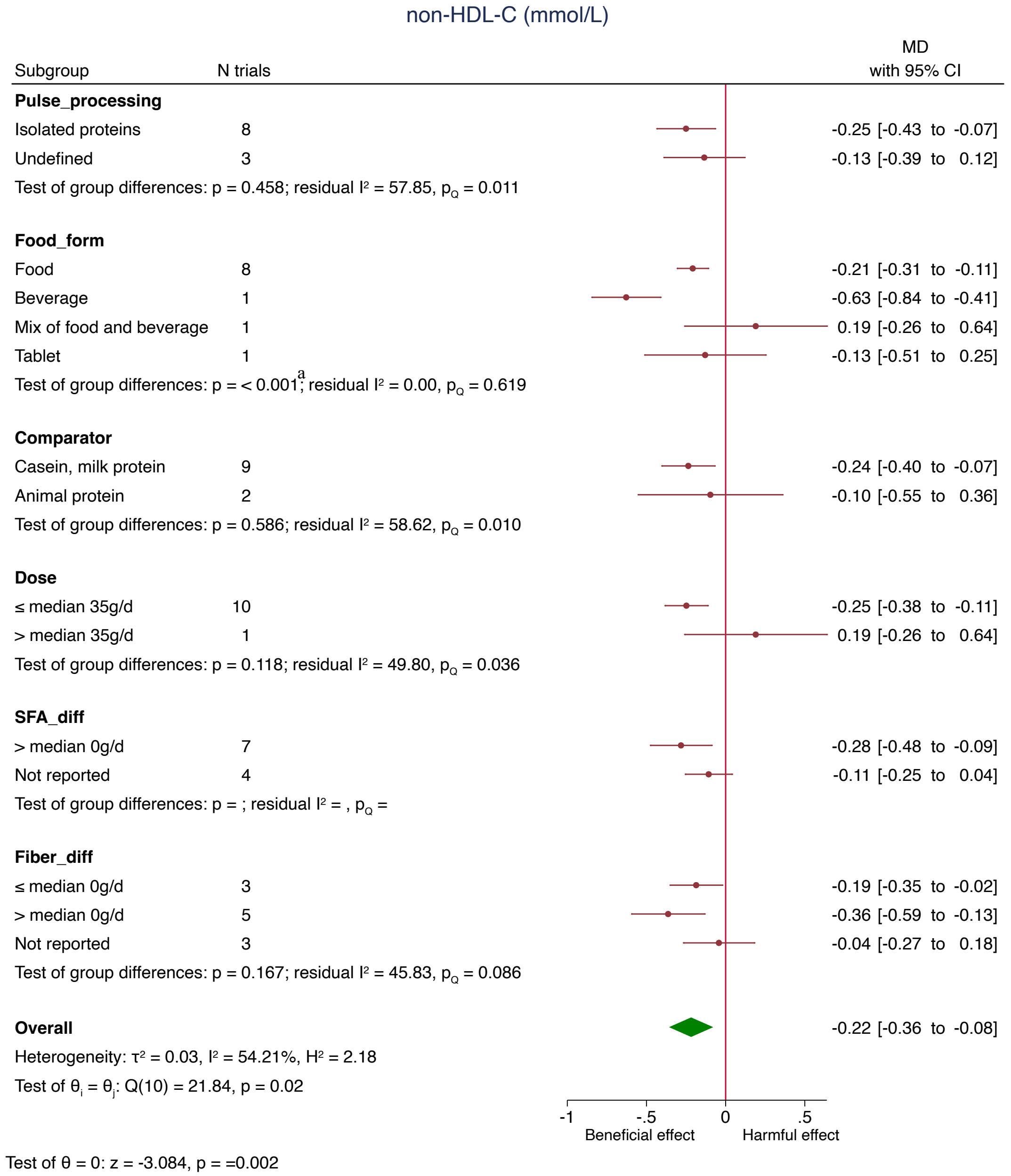

Pooled effect estimates for each subgroup are represented as red circles and the overall effect estimate as the green diamond. Data are expressed as weighted mean differences with 95% confidence intervals using the generic inverse-variance method and random effects DerSimonian-Laird model. Paired analyses were applied to all crossover trials. Inter-study heterogeneity was assessed using the Cochran Q statistic and quantified using the I^2 statistic, with significance set at p<0.100 and $I^2 \geq 50\%$ considered to be evidence of substantial heterogeneity.
The pooled effect summary was calculated with the χ^2 test. The test for subgroup differences was calculated with meta-regression, which uses the Wald test.
SFA_diff refers to the difference in SFA (g/d) of the overall diet between the intervention and control; Fiber_diff refers to the difference in fiber (g/d) of the overall diet between the intervention and control
[a] Pairwise between-subgroup mean differences (95% confidence intervals) for Food form were as follows: Food vs. Beverage -0.42 mmol/L (-0.658,-0.183); Food vs. Mix of food and beverage 0.398 mmol/L (-0.0614, 0.857); Food vs. Tablet 0.0786 mmol/L (-0.316, 0.473); Beverage vs. Mix of food and beverage 0.818 mmol/L (0.32, 1.32); Beverage vs. Tablet 0.499 mmol/L (0.0597, 0.938); Mix of food and beverage vs. Tablet -0.319 mmol/L (-0.909, 0.27)

CI, confidence interval; MD, mean difference; non-HDL-C, non-high density lipoprotein cholesterol

Supplemental Figure S12 (3 of 3). Subgroup analyses for the effect of extracted pulse proteins on non-HDL-C

non-HDL-C (mmol/L)

Subgroup	N trials	MD with 95% CI
**Funding**		
Agency	7	-0.13 [-0.26 to 0.00]
Industry	3	-0.36 [-0.63 to -0.08]
Agency and industry	1	-0.13 [-0.51 to 0.25]
Test of group differences: p = 0.216; residual I^2 = 44.65, p_Q = 0.071		
**Type_of_mean_difference**		
Change from baseline	10	-0.20 [-0.37 to -0.03]
End differences	1	-0.29 [-0.44 to -0.13]
Test of group differences: p = 0.716; residual I^2 = 58.35, p_Q = 0.010		
**Energy_balance**		
Neutral	10	-0.22 [-0.37 to -0.07]
Positive	1	-0.13 [-0.51 to 0.25]
Test of group differences: p = 0.728; residual I^2 = 57.95, p_Q = 0.011		
**Energy_level**		
Substitution	11	-0.22 [-0.36 to -0.08]
Test of group differences: p = ; residual I^2 = , p_Q =		
**Design**		
Parallel	7	-0.14 [-0.30 to 0.01]
Crossover	4	-0.29 [-0.52 to -0.05]
Test of group differences: p = 0.304; residual I^2 = 52.85, p_Q = 0.024		
**Follow_up**		
≤ 12 weeks	11	-0.22 [-0.36 to -0.08]
Test of group differences: p = ; residual I^2 = , p_Q =		
**Feeding_Control**		
Supplemented	11	-0.22 [-0.36 to -0.08]
Test of group differences: p = ; residual I^2 = , p_Q =		
**Overall**		-0.22 [-0.36 to -0.08]
Heterogeneity: τ^2 = 0.03, I^2 = 54.21%, H^2 = 2.18		
Test of $\theta_i = \theta_j$: Q(10) = 21.84, p = 0.02		

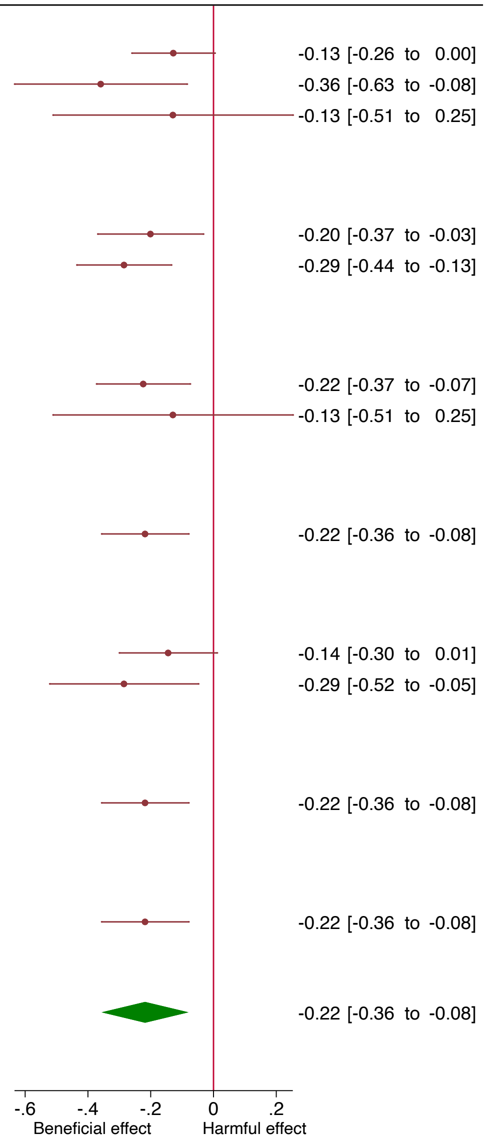

Test of θ = 0: z = -3.084, p = =0.002

Pooled effect estimates for each subgroup are represented as red circles and the overall effect estimate as the green diamond. Data are expressed as weighted mean differences with 95% confidence intervals using the generic inverse-variance method and random effects DerSimonian-Laird model. Paired analyses were applied to all crossover trials. Inter-study heterogeneity was assessed using the Cochran Q statistic and quantified using the I^2 statistic, with significance set at p<0.100 and $I^2 \geq 50\%$ considered to be evidence of substantial heterogeneity.
The pooled effect summary was calculated with the χ^2 test. The test for subgroup differences was calculated with meta-regression, which uses the Wald test.

CI, confidence interval; MD, mean difference; non-HDL-C, non-high density lipoprotein cholesterol

Supplemental Figure S13 (1 of 3). Subgroup analyses for the effect of extracted pulse proteins on HDL-C

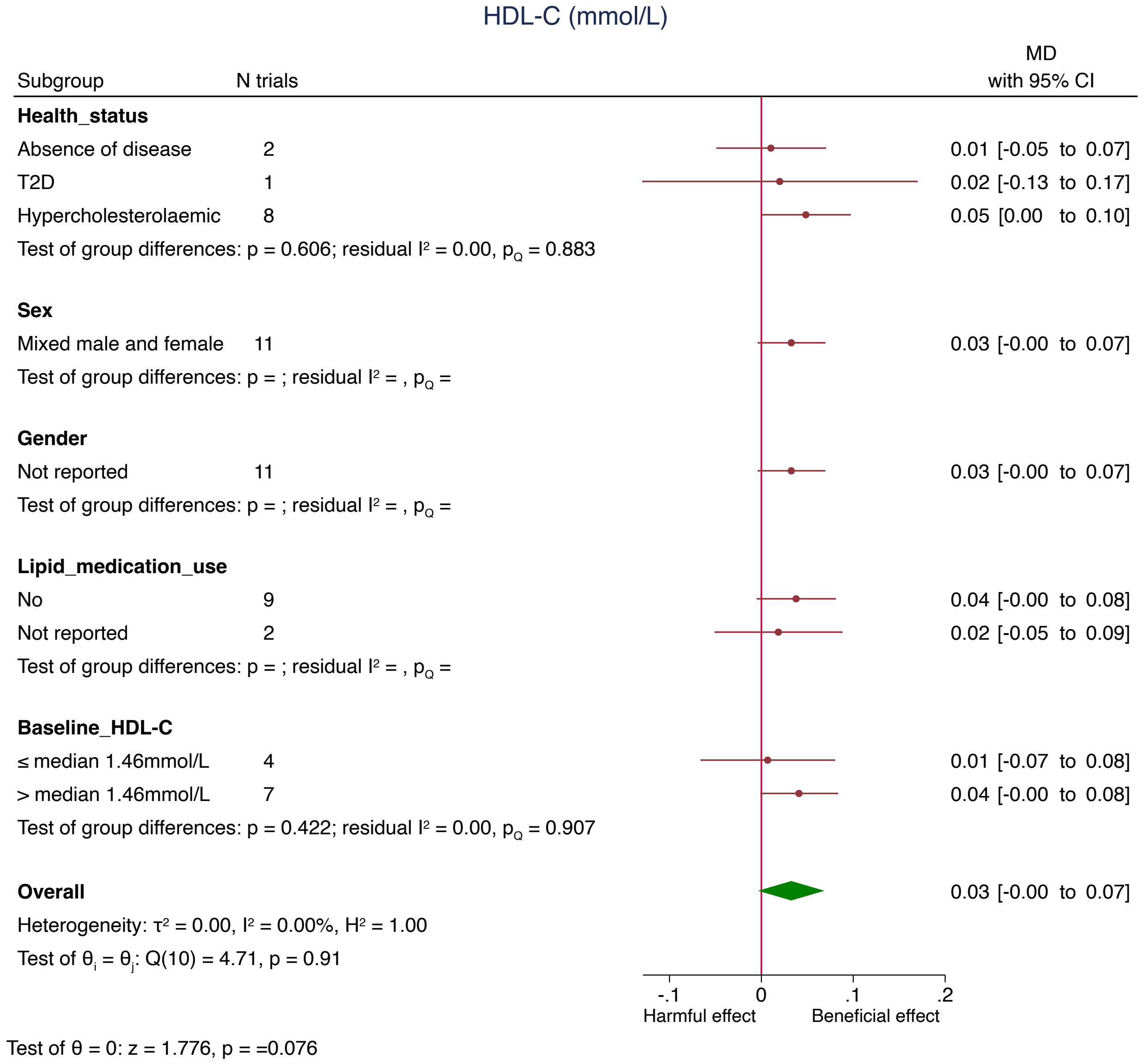

Pooled effect estimates for each subgroup are represented as red circles and the overall effect estimate as the green diamond. Data are expressed as weighted mean differences with 95% confidence intervals using the generic inverse-variance method and random effects DerSimonian-Laird model. Paired analyses were applied to all crossover trials. Inter-study heterogeneity was assessed using the Cochran Q statistic and quantified using the I^2 statistic, with significance set at $p<0.100$ and $I^2 \geq 50\%$ considered to be evidence of substantial heterogeneity.
The pooled effect summary was calculated with the χ^2 test. The test for subgroup differences was calculated with meta-regression, which uses the Wald test.

CI, confidence interval; HDL-C, high density lipoprotein cholesterol; MD, mean difference; T2D, type 2 diabetes

Supplemental Figure S13 (2 of 3). Subgroup analyses for the effect of extracted pulse proteins on HDL-C

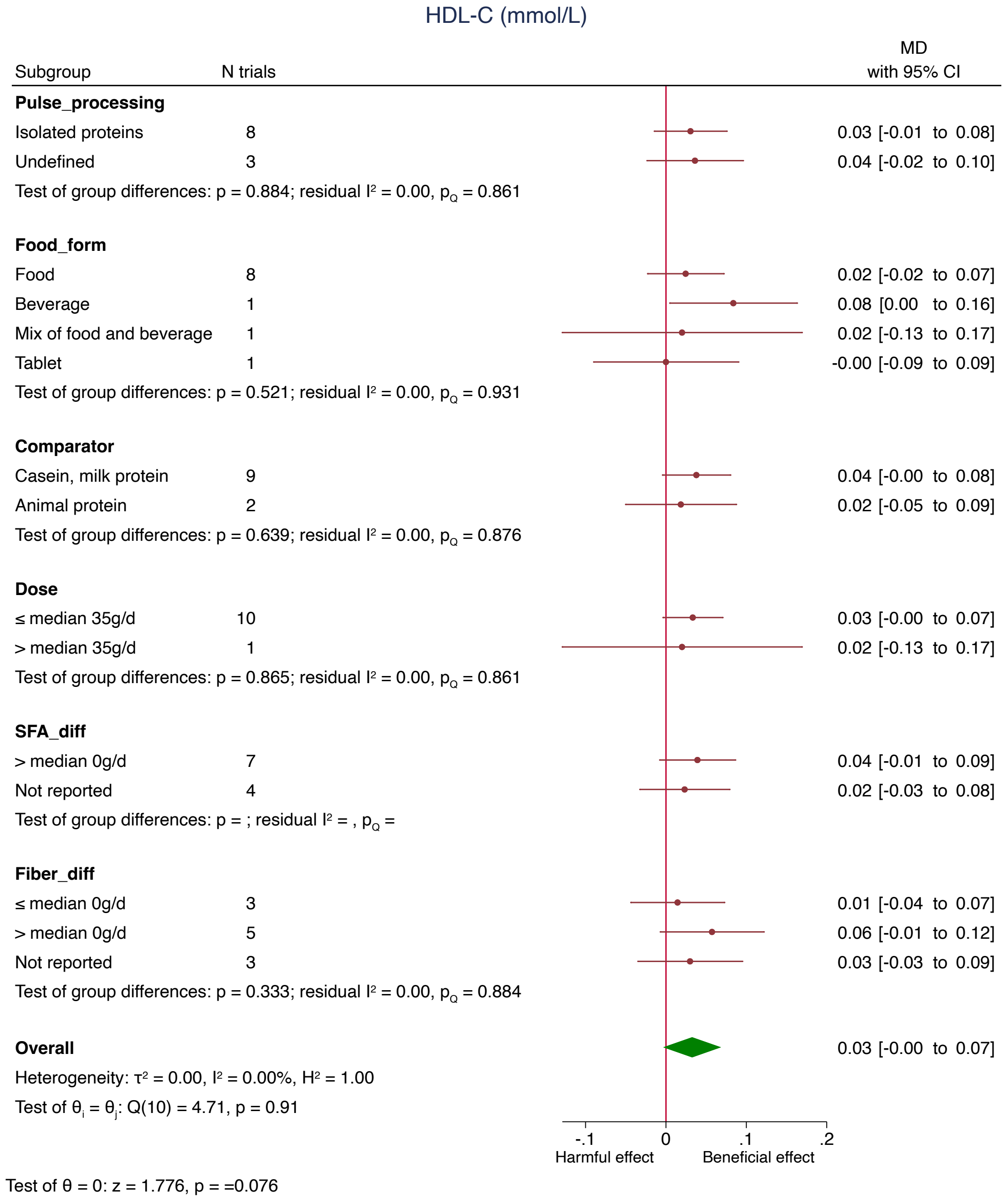

Pooled effect estimates for each subgroup are represented as red circles and the overall effect estimate as the green diamond. Data are expressed as weighted mean differences with 95% confidence intervals using the generic inverse-variance method and random effects DerSimonian-Laird model. Paired analyses were applied to all crossover trials. Inter-study heterogeneity was assessed using the Cochran Q statistic and quantified using the I^2 statistic, with significance set at p<0.100 and $I^2 \geq 50\%$ considered to be evidence of substantial heterogeneity.
The pooled effect summary was calculated with the χ^2 test. The test for subgroup differences was calculated with meta-regression, which uses the Wald test.
SFA_diff refers to the difference in SFA (g/d) of the overall diet between the intervention and control; Fiber_diff refers to the difference in fiber (g/d) of the overall diet between the intervention and control

CI, confidence interval; HDL-C, high density lipoprotein cholesterol; MD, mean difference

Supplemental Figure S13 (3 of 3). Subgroup analyses for the effect of extracted pulse proteins on HDL-C

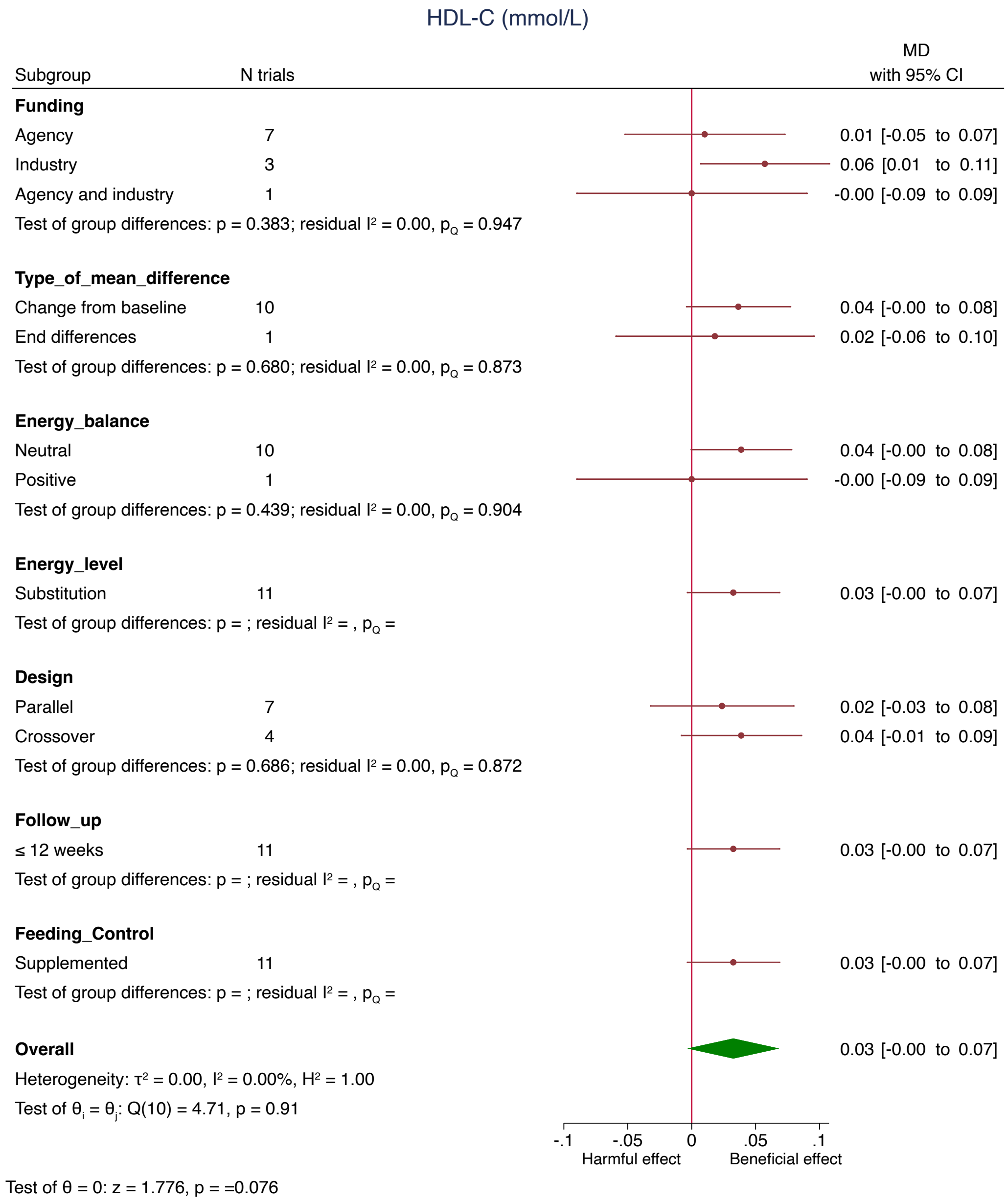

Pooled effect estimates for each subgroup are represented as red circles and the overall effect estimate as the green diamond. Data are expressed as weighted mean differences with 95% confidence intervals using the generic inverse-variance method and random effects DerSimonian-Laird model. Paired analyses were applied to all crossover trials. Inter-study heterogeneity was assessed using the Cochran Q statistic and quantified using the I^2 statistic, with significance set at $p<0.100$ and $I^2 \geq 50\%$ considered to be evidence of substantial heterogeneity.
The pooled effect summary was calculated with the χ^2 test. The test for subgroup differences was calculated with meta-regression, which uses the Wald test.

CI, confidence interval; HDL-C, high density lipoprotein cholesterol; MD, mean difference

Supplemental Figure S14 (1 of 3). Subgroup analyses for the effect of extracted pulse proteins on TG

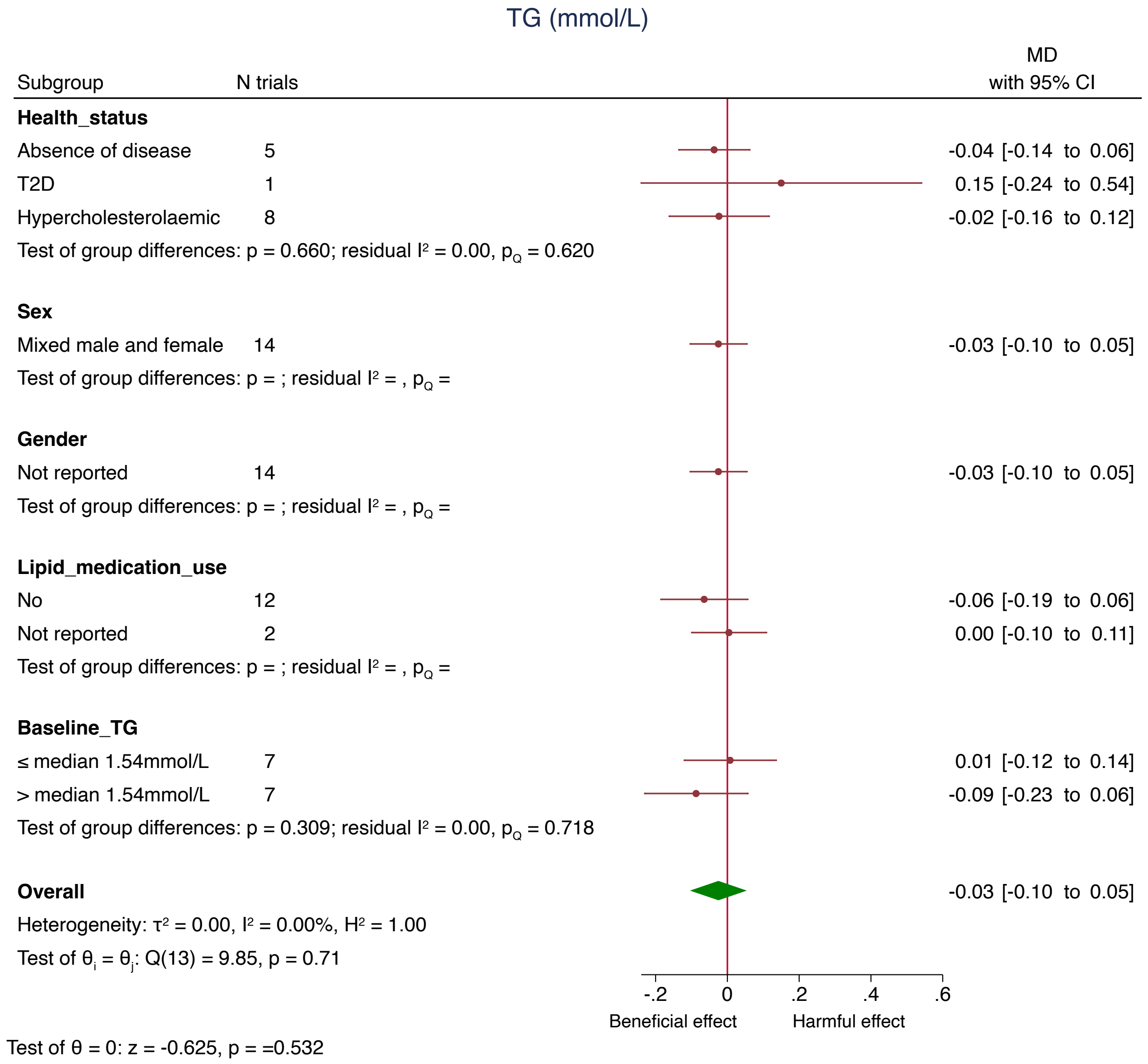

Pooled effect estimates for each subgroup are represented as red circles and the overall effect estimate as the green diamond. Data are expressed as weighted mean differences with 95% confidence intervals using the generic inverse-variance method and random effects DerSimonian-Laird model. Paired analyses were applied to all crossover trials. Inter-study heterogeneity was assessed using the Cochran Q statistic and quantified using the I^2 statistic, with significance set at $p<0.100$ and $I^2 \geq 50\%$ considered to be evidence of substantial heterogeneity.
The pooled effect summary was calculated with the χ^2 test. The test for subgroup differences was calculated with meta-regression, which uses the Wald test.

CI, confidence interval; MD, mean difference; TG, triglyceride

Supplemental Figure S14 (2 of 3). Subgroup analyses for the effect of extracted pulse proteins on TG

TG (mmol/L)

Subgroup	N trials	MD with 95% CI
**Pulse_processing**		
Isolated proteins	11	-0.11 [-0.23 to 0.02]
Undefined	3	0.11 [-0.12 to 0.35]
Test of group differences: p = 0.108; residual I² = 0.00, p_Q = 0.840		
**Food_form**		
Food	8	-0.00 [-0.09 to 0.09]
Beverage	1	-0.12 [-0.37 to 0.14]
Mix of food and beverage	1	0.15 [-0.24 to 0.54]
Tablet	4	-0.19 [-0.44 to 0.05]
Test of group differences: p = 0.341; residual I² = 0.00, p_Q = 0.772		
**Comparator**		
Casein, milk protein	12	-0.06 [-0.19 to 0.06]
Animal protein	2	0.00 [-0.10 to 0.11]
Test of group differences: p = 0.394; residual I² = 0.00, p_Q = 0.692		
**Dose**		
≤ median 35g/d	13	-0.03 [-0.11 to 0.05]
> median 35g/d	1	0.15 [-0.24 to 0.54]
Test of group differences: p = 0.369; residual I² = 0.00, p_Q = 0.699		
**SFA_diff**		
> median 0g/d	7	-0.02 [-0.10 to 0.07]
Not reported	7	-0.06 [-0.25 to 0.14]
Test of group differences: p = ; residual I² = , p_Q =		
**Fiber_diff**		
≤ median 0g/d	3	-0.01 [-0.12 to 0.09]
> median 0g/d	5	-0.07 [-0.24 to 0.09]
Not reported	6	-0.01 [-0.24 to 0.23]
Test of group differences: p = 0.559; residual I² = 0.00, p_Q = 0.953		
**Overall**		-0.03 [-0.10 to 0.05]
Heterogeneity: τ² = 0.00, I² = 0.00%, H² = 1.00		
Test of $\theta_i = \theta_j$: Q(13) = 9.85, p = 0.71		

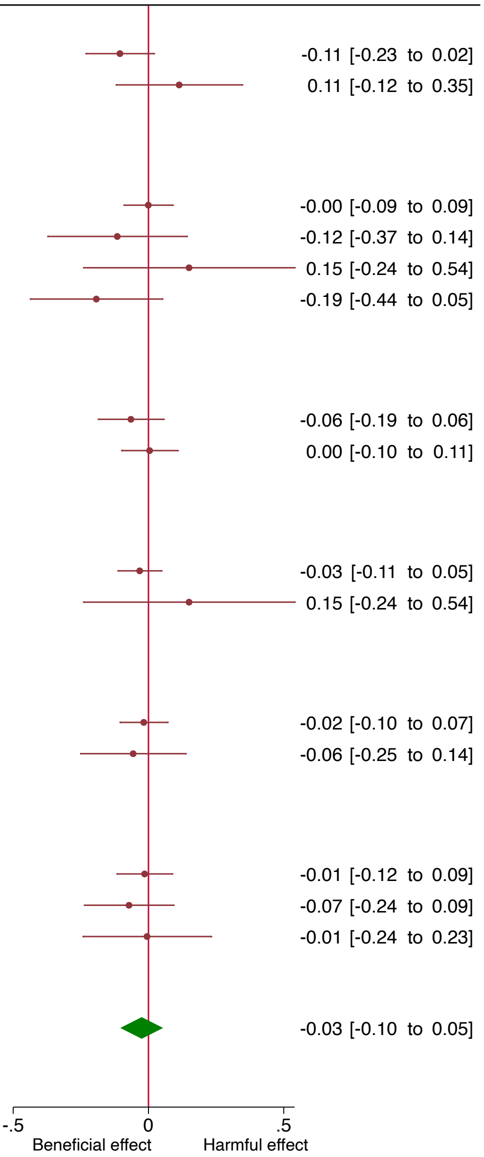

Test of θ = 0: z = -0.625, p = =0.532

Pooled effect estimates for each subgroup are represented as red circles and the overall effect estimate as the green diamond. Data are expressed as weighted mean differences with 95% confidence intervals using the generic inverse-variance method and random effects DerSimonian-Laird model. Paired analyses were applied to all crossover trials. Inter-study heterogeneity was assessed using the Cochran Q statistic and quantified using the I^2 statistic, with significance set at p<0.100 and I^2≥50% considered to be evidence of substantial heterogeneity.
The pooled effect summary was calculated with the χ^2 test. The test for subgroup differences was calculated with meta-regression, which uses the Wald test.
SFA_diff refers to the difference in SFA (g/d) of the overall diet between the intervention and control; Fiber_diff refers to the difference in fiber (g/d) of the overall diet between the intervention and control

CI, confidence interval; MD, mean difference; TG, triglyceride

Supplemental Figure S14 (3 of 3). Subgroup analyses for the effect of extracted pulse proteins on TG

TG (mmol/L)

Subgroup	N trials	MD with 95% CI
**Funding**		
Agency	7	-0.02 [-0.18 to 0.14]
Industry	3	0.03 [-0.17 to 0.23]
Agency and industry	4	-0.19 [-0.44 to 0.05]
Test of group differences: $p = 0.357$; residual $I^2 = 0.00$, $p_Q = 0.732$		
**Type_of_mean_difference**		
Change from baseline	13	-0.05 [-0.16 to 0.07]
End differences	1	-0.01 [-0.11 to 0.10]
Test of group differences: $p = 0.625$; residual $I^2 = 0.00$, $p_Q = 0.650$		
**Energy_balance**		
Neutral	10	-0.01 [-0.09 to 0.08]
Positive	4	-0.19 [-0.44 to 0.05]
Test of group differences: $p = 0.155$; residual $I^2 = 0.00$, $p_Q = 0.799$		
**Energy_level**		
Substitution	14	-0.03 [-0.10 to 0.05]
Test of group differences: p = ; residual I^2 = , p_Q =		
**Design**		
Parallel	10	-0.02 [-0.16 to 0.12]
Crossover	4	-0.03 [-0.12 to 0.07]
Test of group differences: $p = 0.912$; residual $I^2 = 0.00$, $p_Q = 0.630$		
**Follow_up**		
≤ 12 weeks	14	-0.03 [-0.10 to 0.05]
Test of group differences: p = ; residual I^2 = , p_Q =		
**Feeding_Control**		
Supplemented	14	-0.03 [-0.10 to 0.05]
Test of group differences: p = ; residual I^2 = , p_Q =		
**Overall**		-0.03 [-0.10 to 0.05]
Heterogeneity: $\tau^2 = 0.00$, $I^2 = 0.00\%$, $H^2 = 1.00$		
Test of $\theta_i = \theta_j$: $Q(13) = 9.85$, $p = 0.71$		

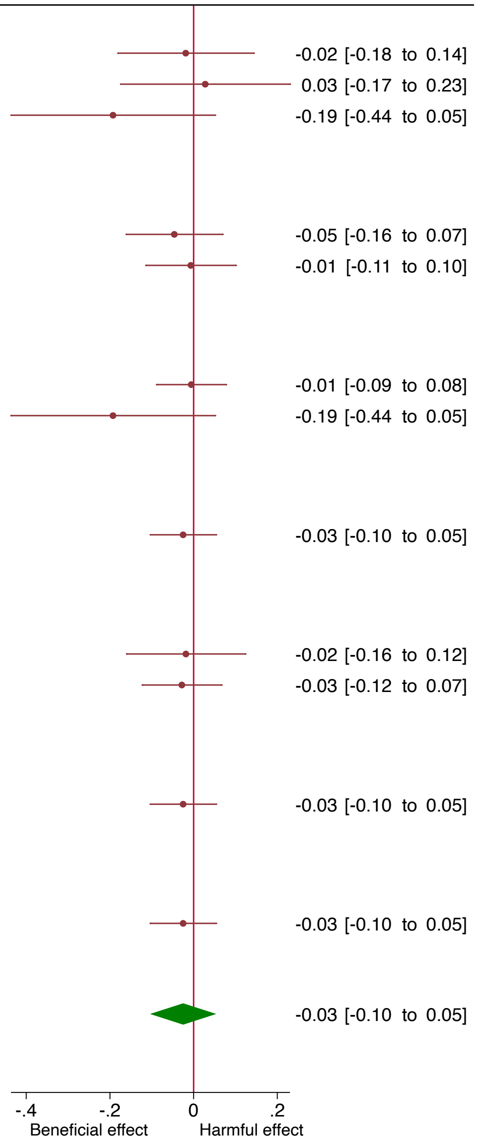

Test of $\theta = 0$: $z = -0.625$, p = =0.532

Pooled effect estimates for each subgroup are represented as red circles and the overall effect estimate as the green diamond. Data are expressed as weighted mean differences with 95% confidence intervals using the generic inverse-variance method and random effects DerSimonian-Laird model. Paired analyses were applied to all crossover trials. Inter-study heterogeneity was assessed using the Cochran Q statistic and quantified using the I^2 statistic, with significance set at $p<0.100$ and $I^2 \geq 50\%$ considered to be evidence of substantial heterogeneity.
The pooled effect summary was calculated with the χ^2 test. The test for subgroup differences was calculated with meta-regression, which uses the Wald test.

CI, confidence interval; MD, mean difference; TG, triglyceride

Supplemental Figure S15. Risk of bias subgroup analyses for the effect of extracted pulse proteins on LDL-C

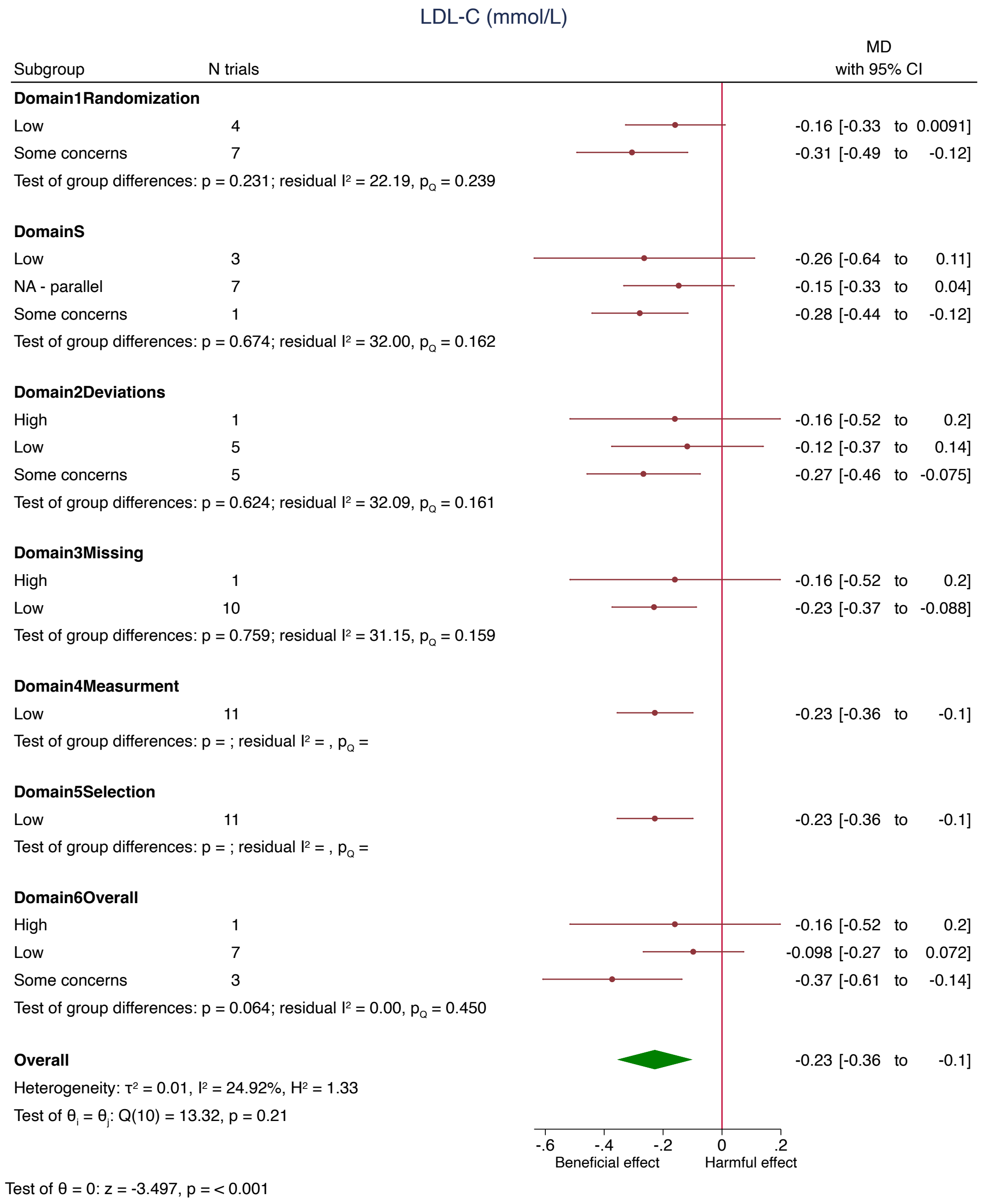

Pooled effect estimates for each subgroup are represented as red circles and the overall effect estimate as the green diamond. Data are expressed as weighted mean differences with 95% confidence intervals using the generic inverse-variance method and random effects DerSimonian-Laird model. Paired analyses were applied to all crossover trials. Inter-study heterogeneity was assessed using the Cochran Q statistic and quantified using the I^2 statistic, with significance set at p<0.100 and $I^2 \geq 50\%$ considered to be evidence of substantial heterogeneity.
The pooled effect summary was calculated with the χ^2 test. The test for subgroup differences was calculated with meta-regression, which uses the Wald test. Note: Domain S refers to the risk of bias arising from period and carryover effects and was only applicable to crossover trials.

LDL-C, low-density lipoprotein cholesterol; MD, mean difference

Supplemental Figure S16. Risk of bias subgroup analyses for the effect of extracted pulse proteins on non-HDL-C

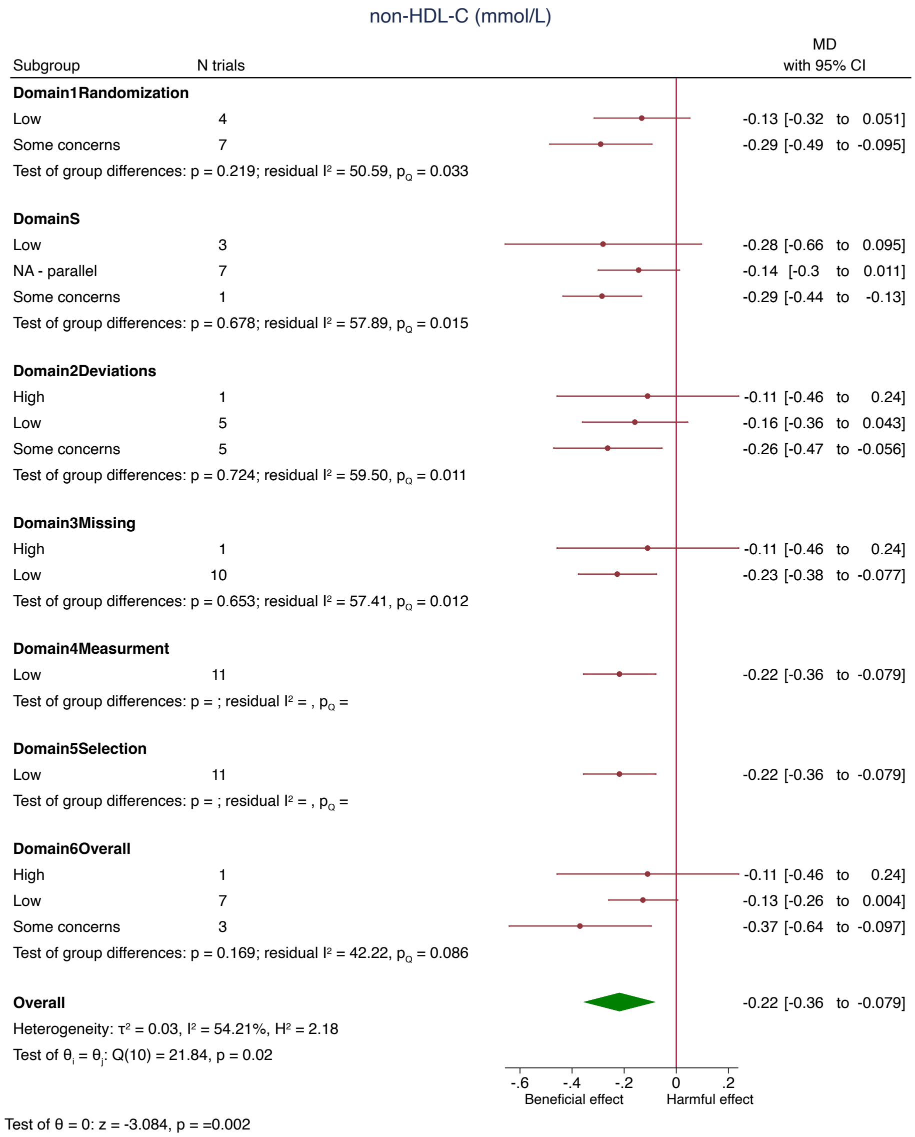

Pooled effect estimates for each subgroup are represented as red circles and the overall effect estimate as the green diamond. Data are expressed as weighted mean differences with 95% confidence intervals using the generic inverse-variance method and random effects DerSimonian-Laird model. Paired analyses were applied to all crossover trials. Inter-study heterogeneity was assessed using the Cochran Q statistic and quantified using the I^2 statistic, with significance set at $p<0.100$ and $I^2 \geq 50\%$ considered to be evidence of substantial heterogeneity.
The pooled effect summary was calculated with the χ^2 test. The test for subgroup differences was calculated with meta-regression, which uses the Wald test. Note: Domain S refers to the risk of bias arising from period and carryover effects and was only applicable to crossover trials.

MD, mean difference; non-HDL-C, non-high density lipoprotein cholesterol

Supplemental Figure S17. Risk of bias subgroup analyses for the effect of extracted pulse proteins on HDL-C

HDL-C (mmol/L)

Subgroup	N trials	MD with 95% CI
**Domain1Randomization**		
Low	4	0.015 [-0.039 to 0.069]
Some concerns	7	0.046 [-0.0018 to 0.094]
Test of group differences: p = 0.400; residual I² = 0.00, p_Q = 0.911		
**DomainS**		
Low	3	0.048 [-0.016 to 0.11]
NA - parallel	7	0.024 [-0.032 to 0.08]
Some concerns	1	0.018 [-0.059 to 0.095]
Test of group differences: p = 0.743; residual I² = 0.00, p_Q = 0.847		
**Domain2Deviations**		
High	1	0.09 [-0.03 to 0.21]
Low	5	0.011 [-0.078 to 0.099]
Some concerns	5	0.03 [-0.011 to 0.072]
Test of group differences: p = 0.571; residual I² = 0.00, p_Q = 0.892		
**Domain3Missing**		
High	1	0.09 [-0.03 to 0.21]
Low	10	0.027 [-0.011 to 0.065]
Test of group differences: p = 0.326; residual I² = 0.00, p_Q = 0.927		
**Domain4Measurment**		
Low	11	0.033 [-0.0034 to 0.068]
Test of group differences: p = ; residual I² = , p_Q =		
**Domain5Selection**		
Low	11	0.033 [-0.0034 to 0.068]
Test of group differences: p = ; residual I² = , p_Q =		
**Domain6Overall**		
High	1	0.09 [-0.03 to 0.21]
Low	7	0.01 [-0.052 to 0.073]
Some concerns	3	0.036 [-0.013 to 0.086]
Test of group differences: p = 0.497; residual I² = 0.00, p_Q = 0.913		
**Overall**		0.033 [-0.0034 to 0.068]
Heterogeneity: τ^2 = 0.00, I² = 0.00%, H² = 1.00		
Test of $\theta_i = \theta_j$: Q(10) = 4.71, p = 0.91		

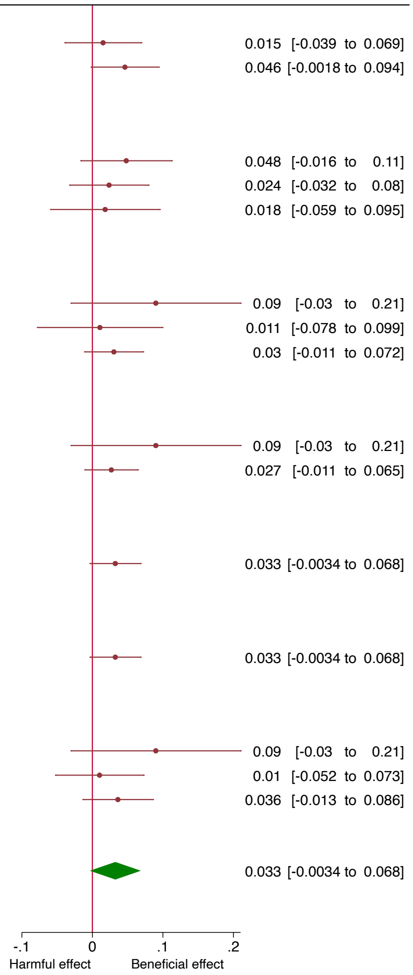

Test of θ = 0: z = 1.776, p = =0.076

Pooled effect estimates for each subgroup are represented as red circles and the overall effect estimate as the green diamond. Data are expressed as weighted mean differences with 95% confidence intervals using the generic inverse-variance method and random effects DerSimonian-Laird model. Paired analyses were applied to all crossover trials. Inter-study heterogeneity was assessed using the Cochran Q statistic and quantified using the I^2 statistic, with significance set at p<0.100 and $I^2 \geq 50\%$ considered to be evidence of substantial heterogeneity.
The pooled effect summary was calculated with the χ^2 test. The test for subgroup differences was calculated with meta-regression, which uses the Wald test. Note: Domain S refers to the risk of bias arising from period and carryover effects and was only applicable to crossover trials.

HDL-C, high density lipoprotein cholesterol; MD, mean difference

Supplemental Figure S18. Risk of bias subgroup analyses for the effect of extracted pulse proteins on TG

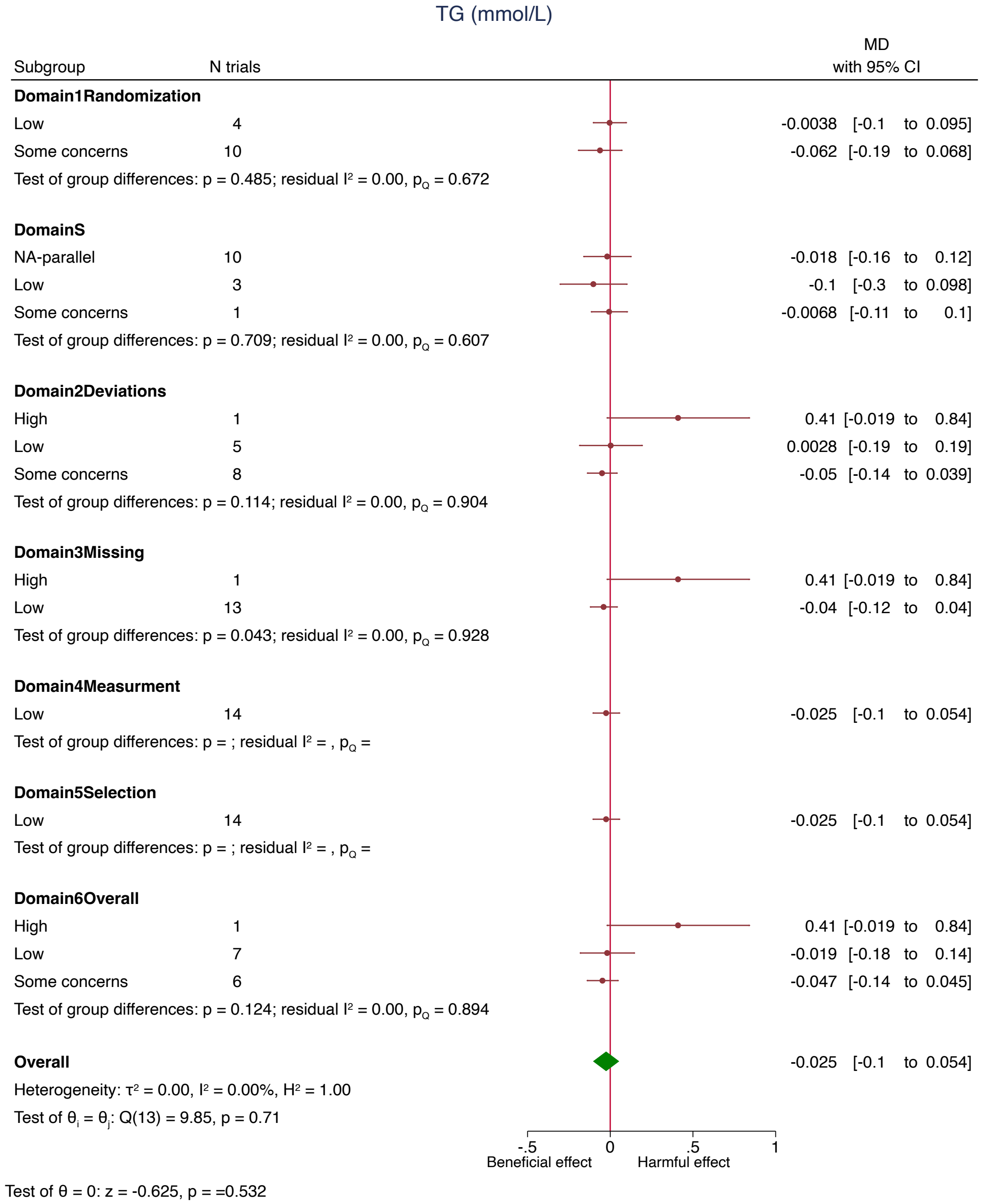

Pooled effect estimates for each subgroup are represented as red circles and the overall effect estimate as the green diamond. Data are expressed as weighted mean differences with 95% confidence intervals using the generic inverse-variance method and random effects DerSimonian-Laird model. Paired analyses were applied to all crossover trials. Inter-study heterogeneity was assessed using the Cochran Q statistic and quantified using the I^2 statistic, with significance set at $p<0.100$ and $I^2 \geq 50\%$ considered to be evidence of substantial heterogeneity.
The pooled effect summary was calculated with the χ^2 test. The test for subgroup differences was calculated with meta-regression, which uses the Wald test. Note: Domain S refers to the risk of bias arising from period and carryover effects and was only applicable to crossover trials.

MD, mean difference; TG, triglyceride

Supplemental Figure S19. Continuous meta-regression analysis for the effect of extracted pulse proteins on LDL-C*

Continuous meta-regression
LDL-C

Subgroup	Range	Trials	Beta [95% CI]	P_{MD}	Residual I^2 (%)	P_Q
Baseline LDL-C (mmol/L)	3.12— 4.93	11	-0.08 [-0.28 to 0.11]	0.404	28	0.188
Female proportion	0.35— 0.84	11	-1.53 [-2.47 to -0.59]	0.001	0	0.959
Male proportion	0.16— 0.65	11	1.53 [0.59 to 2.47]	0.001	0	0.959
Pulse protein dose (g/d)	5.30—122.26	11	0.00 [-0.00 to 0.01]	0.215	22	0.241
Pulse fiber dose (g/d)	2.64—10.50	6	0.01 [-0.05 to 0.06]	0.733	0	0.798
Mean age (years)	42.20—64.33	11	0.00 [-0.02 to 0.03]	0.826	32	0.149
Follow-up (weeks)	4.00— 8.00	11	-0.04 [-0.11 to 0.04]	0.377	25	0.209
Difference in SFA of the overall diet between the interventions (g/d)	0.00— 7.00	7	0.01 [-0.06 to 0.09]	0.717	42	0.127
Difference in fiber of the overall diet between the interventions (g/d)	-5.60— 0.10	8	-0.00 [-0.09 to 0.08]	0.970	45	0.093

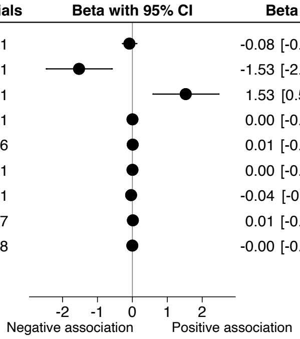

Data is presented as between group mean difference (95% CI) for a 1-unit change in the predictor variable. β –coefficients were estimated using continuous meta-regression analysis. A positive β -coefficient implies an increase in outcome in the isoflavone intervention as the subgroup variable increases, and a negative β -coefficient implies a decrease in outcome. Residual I^2 reports inter-study heterogeneity not explained by the subgroup and was estimated using the Cochran Q statistic.

CI, confidence interval; LDL-C, low-density lipoprotein cholesterol; SFA, saturated fatty acid

*N=5 trial comparisons did not report fiber dose consumed per day, N=4 did not report difference in SFA of overall diet between interventions, N=3 did not report difference in fiber of overall diet between interventions

Supplemental Figure S20. Continuous meta-regression analysis for the effect of extracted pulse proteins on non-HDL-C*

Continuous meta-regression

non-HDL-C

Subgroup	Range	Trials	Beta [95% CI]	P_{MD}	Residual I^2 (%)	P_Q
Baseline non-HDL-C (mmol/L)	3.65— 5.65	11	-0.10 [-0.29 to 0.08]	0.269	54	0.020
Female proportion	0.35— 0.84	11	-1.60 [-2.36 to -0.84]	<0.001	0	0.851
Male proportion	0.16— 0.65	11	1.60 [0.84 to 2.36]	<0.001	0	0.851
Pulse protein dose (g/d)	5.30—122.26	11	0.00 [-0.00 to 0.01]	0.164	52	0.028
Pulse fiber dose (g/d)	2.64—10.50	6	-0.00 [-0.04 to 0.04]	0.985	0	0.741
Mean age (years)	42.20—64.33	11	0.00 [-0.02 to 0.03]	0.898	59	0.010
Follow-up (weeks)	4.00— 8.00	11	-0.02 [-0.11 to 0.08]	0.718	57	0.014
Difference in SFA of the overall diet between the interventions (g/d)	0.00— 7.00	7	0.02 [-0.06 to 0.10]	0.607	62	0.023
Difference in fiber of the overall diet between the interventions (g/d)	-5.60— 0.10	8	-0.01 [-0.10 to 0.08]	0.884	63	0.013

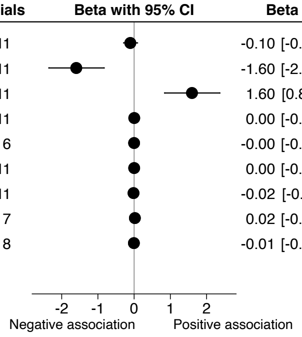

Data is presented as between group mean difference (95% CI) for a 1-unit change in the predictor variable. β –coefficients were estimated using continuous meta-regression analysis. A positive β -coefficient implies an increase in outcome in the isoflavone intervention as the subgroup variable increases, and a negative β -coefficient implies a decrease in outcome. Residual I^2 reports inter-study heterogeneity not explained by the subgroup and was estimated using the Cochran Q statistic.

CI, confidence interval; non-HDL-C, non-high density lipoprotein cholesterol; SFA, saturated fatty acid

*N=5 trial comparisons did not report fiber dose consumed per day, N=4 did not report difference in saturated fatty acid (SFA) of overall diet between interventions, N=3 did not report difference in fiber of overall diet between interventions

Supplemental Figure S21. Continuous meta-regression analysis for the effect of extracted pulse proteins on HDL-C*

Continuous meta-regression

HDL-C

Subgroup	Range	Trials	Beta [95% CI]	P_{MD}	Residual I^2 (%)	P_Q
Baseline HDL-C (mmol/L)	1.11— 1.73	11	0.12 [-0.13 to 0.37]	0.347	0	0.923
Female proportion	0.35— 0.84	11	0.15 [-0.12 to 0.43]	0.277	0	0.940
Male proportion	0.16— 0.65	11	-0.15 [-0.43 to 0.12]	0.277	0	0.940
Pulse protein dose (g/d)	5.30—122.26	11	0.00 [-0.00 to 0.00]	0.952	0	0.859
Pulse fiber dose (g/d)	2.64—10.50	6	0.00 [-0.02 to 0.02]	0.985	0	0.985
Mean age (years)	42.20—64.33	11	0.00 [-0.00 to 0.01]	0.765	0	0.866
Follow-up (weeks)	4.00— 8.00	11	-0.00 [-0.02 to 0.02]	0.912	0	0.860
Difference in SFA of the overall diet between the interventions (g/d)	0.00— 7.00	7	-0.01 [-0.02 to 0.01]	0.444	0	0.900
Difference in fiber of the overall diet between the interventions (g/d)	-5.60— 0.10	8	0.01 [-0.01 to 0.02]	0.547	0	0.817

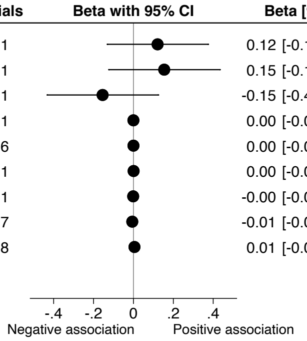

Data is presented as between group mean difference (95% CI) for a 1-unit change in the predictor variable. β –coefficients were estimated using continuous meta-regression analysis. A positive β -coefficient implies an increase in outcome in the isoflavone intervention as the subgroup variable increases, and a negative β -coefficient implies a decrease in outcome. Residual I^2 reports inter-study heterogeneity not explained by the subgroup and was estimated using the Cochran Q statistic.

CI, confidence interval; HDL-C, high density lipoprotein cholesterol; SFA, saturated fatty acid

*N=5 trial comparisons did not report fiber dose consumed per day, N=4 did not report difference in saturated fatty acid (SFA) of overall diet between interventions, N=3 did not report difference in fiber of overall diet between interventions

Supplemental Figure S22. Continuous meta-regression analysis for the effect of extracted pulse proteins on TG*

Continuous meta-regression

TG

Subgroup	Range	Trials	Beta [95% CI]	P_{MD}	Residual I^2 (%)	P_Q
Baseline TG (mmol/L)	1.12— 1.78	14	-0.10 [-0.40 to 0.20]	0.511	0	0.667
Female proportion	0.35— 0.84	14	-0.16 [-0.87 to 0.55]	0.657	0	0.646
Male proportion	0.16— 0.65	14	0.16 [-0.55 to 0.87]	0.657	0	0.646
Pulse protein dose (g/d)	1.32—122.26	14	0.00 [-0.00 to 0.01]	0.134	0	0.815
Pulse fiber dose (g/d)	2.64—10.50	9	0.01 [-0.03 to 0.05]	0.738	0	0.743
Mean age (years)	42.20—64.33	14	0.00 [-0.01 to 0.02]	0.777	0	0.636
Follow-up (weeks)	4.00— 8.00	14	0.01 [-0.03 to 0.06]	0.637	0	0.648
Difference in SFA of the overall diet between the interventions (g/d)	0.00— 7.00	7	0.01 [-0.02 to 0.04]	0.575	0	0.815
Difference in fiber of the overall diet between the interventions (g/d)	-5.60— 0.10	8	-0.01 [-0.05 to 0.02]	0.469	0	0.965

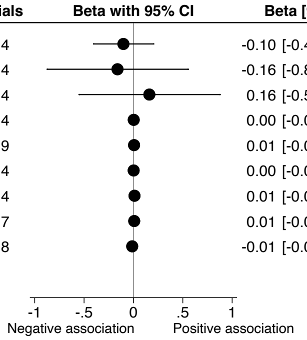

Data is presented as between group mean difference (95% CI) for a 1-unit change in the predictor variable. β –coefficients were estimated using continuous meta-regression analysis. A positive β -coefficient implies an increase in outcome in the isoflavone intervention as the subgroup variable increases, and a negative β -coefficient implies a decrease in outcome. Residual I^2 reports inter-study heterogeneity not explained by the subgroup and was estimated using the Cochran Q statistic.

CI, confidence interval; TG, triglyceride; SFA, saturated fatty acid

*N=5 trial comparisons did not report fiber dose consumed per day, N=7 did not report difference in saturated fatty acid (SFA) of overall diet between interventions, N=6 did not report difference in fiber of overall diet between interventions

Supplemental Figure S23. Continuous meta-regression analysis for the effect of extracted pulse proteins on LDL-C* (with Frota et al. 2015 removed)

Continuous meta-regression

LDL-C

Subgroup	Range	Trials	Beta [95% CI]	P_{MD}	Residual I^2 (%)	P_Q
Baseline LDL-C (mmol/L)	3.12— 4.93	10	0.07 [-0.11 to 0.24]	0.467	0	0.754
Proportion of female	0.35— 0.67	10	-1.23 [-2.70 to 0.24]	0.101	0	0.943
Proportion of male	0.33— 0.65	10	1.23 [-0.24 to 2.70]	0.101	0	0.943
Pulse protein dose (g/d)	5.30—122.26	10	0.00 [-0.00 to 0.01]	0.370	0	0.783
Pulse fiber dose (g/d)	2.64—10.50	6	0.01 [-0.05 to 0.06]	0.733	0	0.798
Mean age (years)	42.20—64.33	10	0.01 [-0.01 to 0.03]	0.238	0	0.842
Follow-up (weeks)	4.00— 8.00	10	-0.04 [-0.10 to 0.02]	0.202	0	0.863
Difference in SFA of the overall diet between the interventions (g/d)	0.00— 7.00	6	-0.02 [-0.06 to 0.03]	0.475	0	0.533
Difference in fiber of the overall diet between the interventions (g/d)	-5.60— 0.00	7	0.03 [-0.02 to 0.08]	0.231	0	0.765

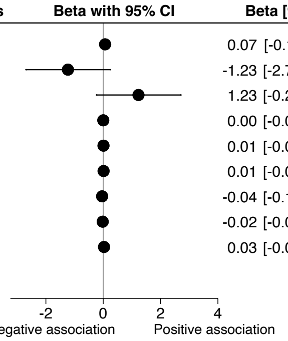

Data is presented as between group mean difference (95% CI) for a 1-unit change in the predictor variable. β –coefficients were estimated using continuous meta-regression analysis. A positive β -coefficient implies an increase in outcome in the isoflavone intervention as the subgroup variable increases, and a negative β -coefficient implies a decrease in outcome. Residual I^2 reports inter-study heterogeneity not explained by the subgroup and was estimated using the Cochran Q statistic.

CI, confidence interval; LDL-C, low-density lipoprotein cholesterol; SFA, saturated fatty acid

*N=4 trial comparisons did not report fiber dose consumed per day, N=4 did not report difference in SFA of overall diet between interventions, N=3 did not report difference in fiber of overall diet between interventions

Supplemental Figure S24. Continuous meta-regression analysis for the effect of extracted pulse proteins on non-HDL-C* (with Frota et al. 2015 removed)

Continuous meta-regression
non-HDL-C

Subgroup	Range	Trials	Beta [95% CI]	P_{MD}	Residual I^2 (%)	P_Q
Baseline non-HDL-C (mmol/L)	3.65— 5.65	10	0.03 [-0.09 to 0.15]	0.639	1	0.427
Female proportion	0.35— 0.67	10	-1.27 [-2.53 to -0.01]	0.048	0	0.818
Male proportion	0.33— 0.65	10	1.27 [0.01 to 2.53]	0.048	0	0.818
Pulse protein dose (g/d)	5.30—122.26	10	0.00 [-0.00 to 0.01]	0.239	0	0.545
Pulse fiber dose (g/d)	2.64—10.50	6	-0.00 [-0.04 to 0.04]	0.985	0	0.741
Mean age (years)	42.20—64.33	10	0.01 [-0.01 to 0.03]	0.248	0	0.539
Follow-up (weeks)	4.00— 8.00	10	-0.03 [-0.08 to 0.02]	0.293	0	0.515
Difference in SFA of the overall diet between the interventions (g/d)	0.00— 7.00	6	-0.01 [-0.06 to 0.05]	0.847	20	0.288
Difference in fiber of the overall diet between the interventions (g/d)	-5.60— 0.00	7	0.02 [-0.02 to 0.06]	0.271	0	0.580

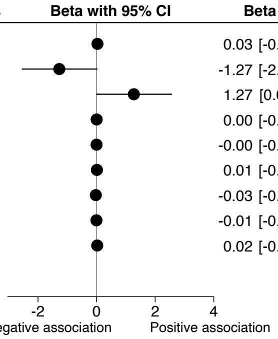

Data is presented as between group mean difference (95% CI) for a 1-unit change in the predictor variable. β –coefficients were estimated using continuous meta-regression analysis. A positive β -coefficient implies an increase in outcome in the isoflavone intervention as the subgroup variable increases, and a negative β -coefficient implies a decrease in outcome. Residual I^2 reports inter-study heterogeneity not explained by the subgroup and was estimated using the Cochran Q statistic.

CI, confidence interval; non-HDL-C, non-high density lipoprotein cholesterol; SFA, saturated fatty acid

*N=4 trial comparisons did not report fiber dose consumed per day, N=4 did not report difference in saturated fatty acid (SFA) of overall diet between interventions, N=3 did not report difference in fiber of overall diet between interventions

Supplemental Figure S25. Linear and non-linear meta-regression analysis for the effect of extracted pulse proteins on LDL-C

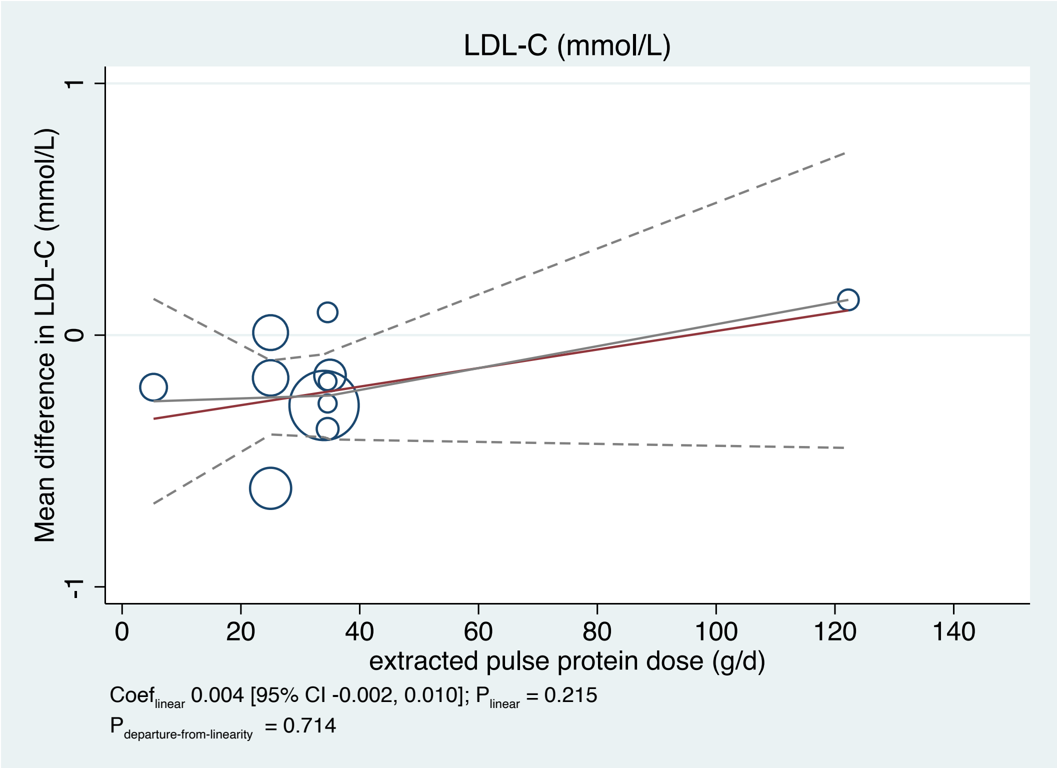

Individual studies are represented by the circles, with their weight in the overall analysis represented by the size of the circles. The straight red line represents the estimate linear dose response and the grey line the non-linear dose response for the extracted pulse dose and the dashed lines represent the upper and lower 95% confidence intervals.

CI, confidence interval; LDL-C, low density lipoprotein cholesterol

Supplemental Figure S26. Linear and non-linear meta-regression analysis for the effect of extracted pulse proteins on non-HDL-C

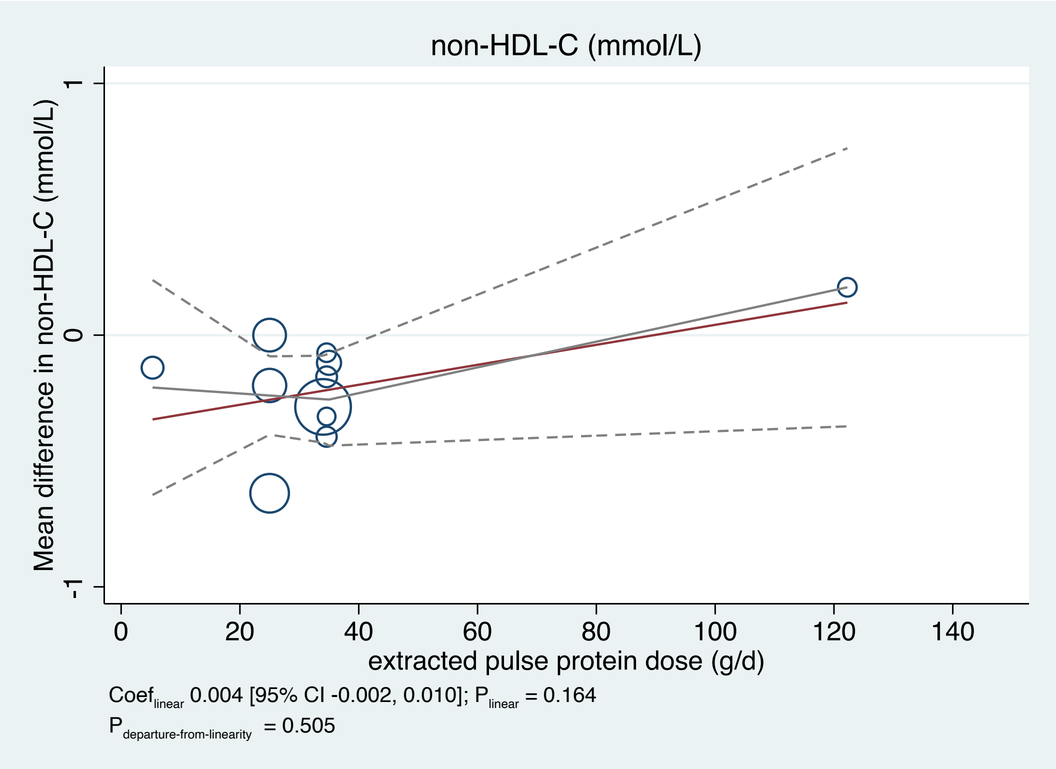

Individual studies are represented by the circles, with their weight in the overall analysis represented by the size of the circles. The straight red line represents the estimate linear dose response and the grey line the non-linear dose response for the extracted pulse dose and the dashed lines represent the upper and lower 95% confidence intervals.

CI, confidence interval; non-HDL-C, non-high density lipoprotein cholesterol

Supplemental Figure S27. Linear and non-linear meta-regression analysis for the effect of extracted pulse proteins on HDL-C

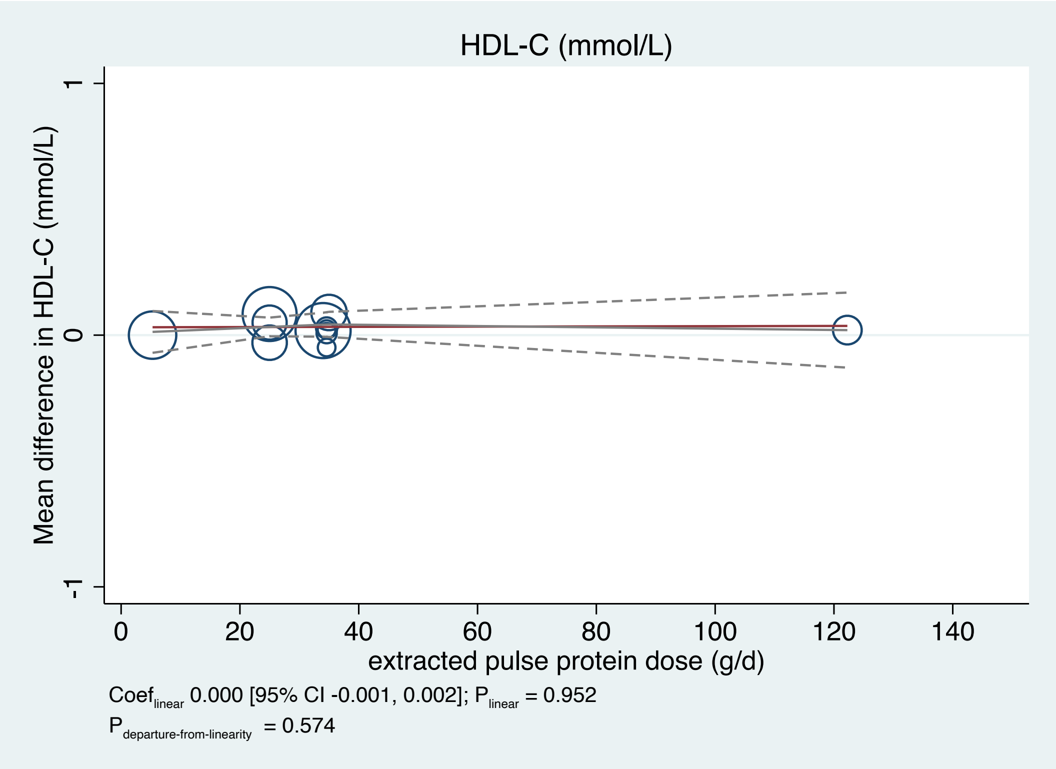

Individual studies are represented by the circles, with their weight in the overall analysis represented by the size of the circles. The straight red line represents the estimate linear dose response and the grey line the non-linear dose response for the extracted pulse dose and the dashed lines represent the upper and lower 95% confidence intervals.

CI, confidence interval; HDL-C, high density lipoprotein cholesterol

Supplemental Figure S28. Linear and non-linear meta-regression analysis for the effect of extracted pulse proteins on TG

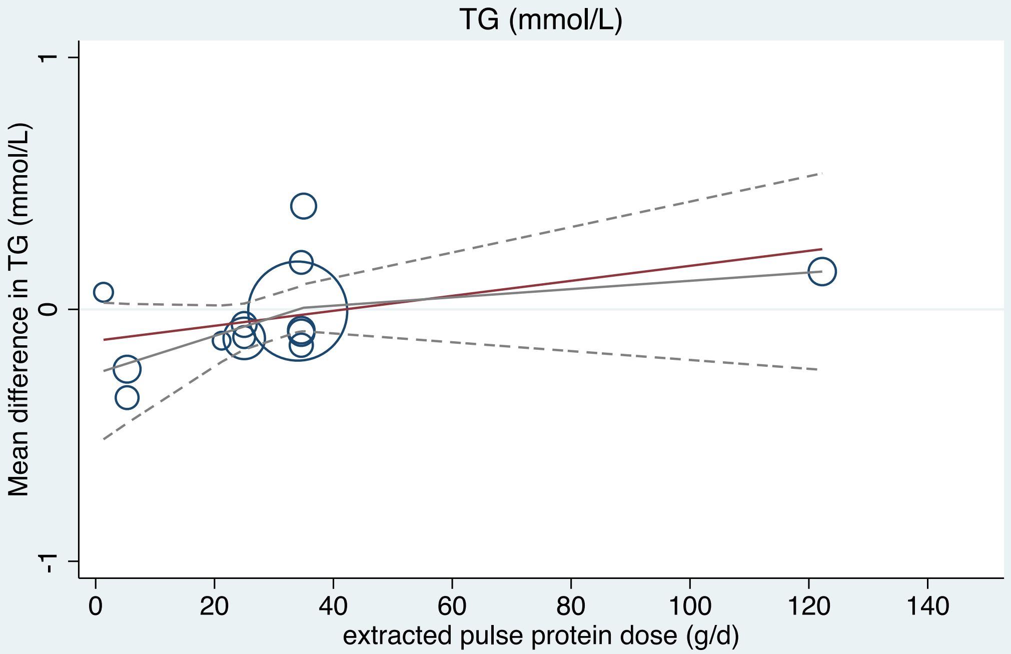

$Coef_{linear}$ 0.003 [95% CI -0.001, 0.007]; P_{linear} = 0.134

$P_{departure-from-linearity}$ = 0.286

Individual studies are represented by the circles, with their weight in the overall analysis represented by the size of the circles. The straight red line represents the estimate linear dose response and the grey line the non-linear dose response for the extracted pulse dose and the dashed lines represent the upper and lower 95% confidence intervals.

CI, confidence interval; TG, triglyceride

Supplemental Figure S29. Publication bias funnel plots for the effect of extracted pulse proteins on LDL-C

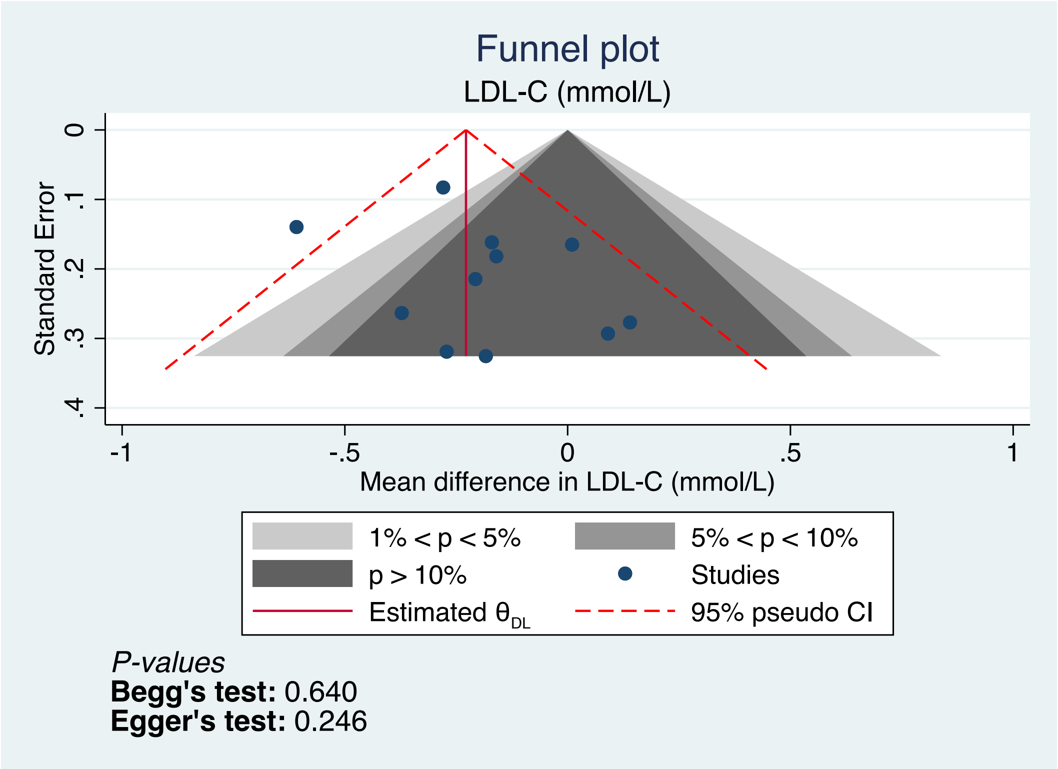

Contour-enhanced funnel plot is a scatter-plot of each trial comparison weighted mean difference (MD) on the x-axis with the standard error (SE) representing precision on the y-axis. The vertical solid red line represents the pooled effect estimate and the dashed red lines represent the pseudo-95% confidence limits. The blue dots represent individual trial comparisons. The contour regions define the regions for the test of significance of individual study effect size for a given p-value range >0.1 (dark grey), 0.5 to <0.1 (medium grey), 0.01 to <0.5 (light grey), <0.01 (white)]. The contour-enhanced funnel plots may suggest funnel-plot asymmetry is due to publication bias when less precise (smaller) studies are missing in the non-significant regions. Quantitative assessment of publication bias was also performed using Egger's and Begg's tests set at a significance level of p<0.1.

CI, confidence interval; LDL-C, low density lipoprotein cholesterol

Supplemental Figure S30. Publication bias funnel plots for the effect of extracted pulse proteins on non-HDL-C

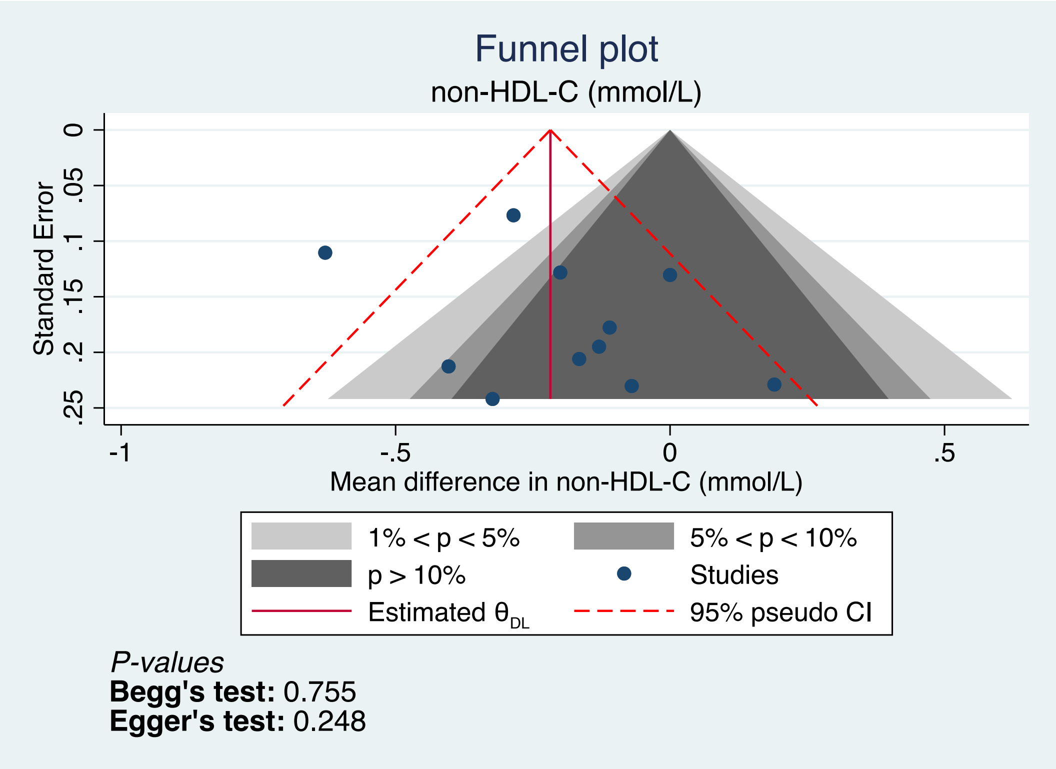

Contour-enhanced funnel plot is a scatter-plot of each trial comparison weighted mean difference (MD) on the x-axis with the standard error (SE) representing precision on the y-axis. The vertical solid red line represents the pooled effect estimate and the dashed red lines represent the pseudo-95% confidence limits. The blue dots represent individual trial comparisons. The contour regions define the regions for the test of significance of individual study effect size for a given p-value range >0.1 (dark grey), 0.5 to <0.1 (medium grey), 0.01 to <0.5 (light grey), <0.01 (white)]. The contour-enhanced funnel plots may suggest funnel-plot asymmetry is due to publication bias when less precise (smaller) studies are missing in the non-significant regions. Quantitative assessment of publication bias was also performed using Egger's and Begg's tests set at a significance level of p<0.1.

CI, confidence interval; non-HDL-C, non-high density lipoprotein cholesterol

Supplemental Figure S31. Publication bias funnel plots for the effect of extracted pulse proteins on HDL-C

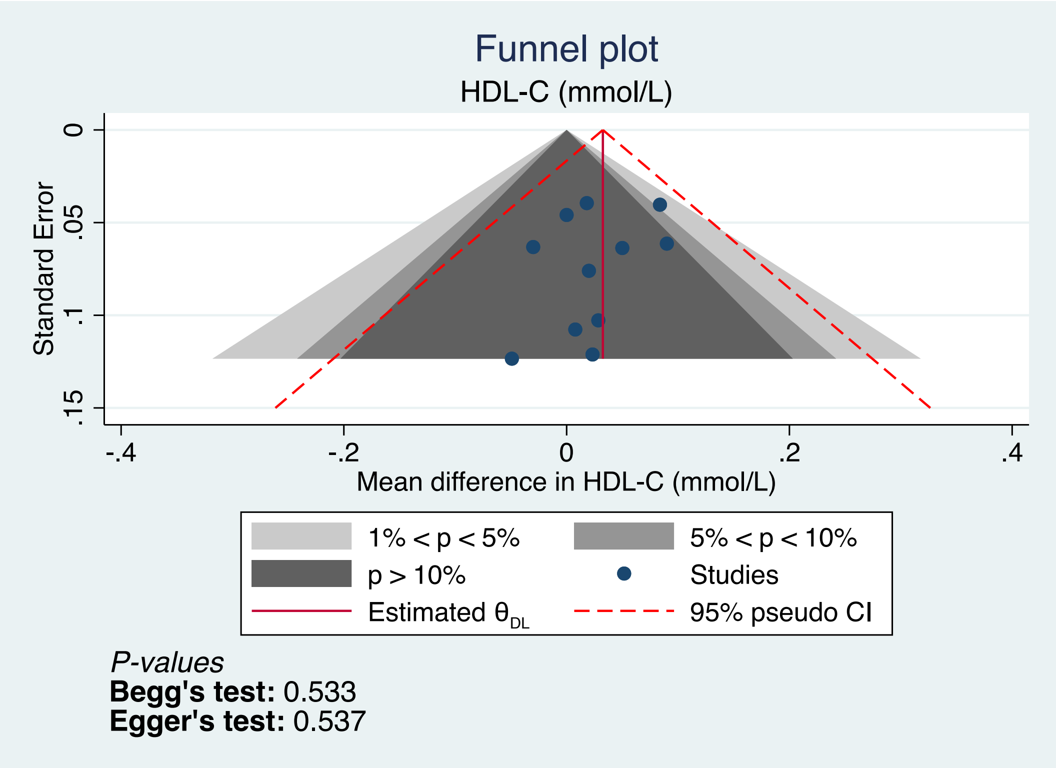

Contour-enhanced funnel plot is a scatter-plot of each trial comparison weighted mean difference (MD) on the x-axis with the standard error (SE) representing precision on the y-axis. The vertical solid red line represents the pooled effect estimate and the dashed red lines represent the pseudo-95% confidence limits. The blue dots represent individual trial comparisons. The contour regions define the regions for the test of significance of individual study effect size for a given p-value range >0.1 (dark grey), 0.5 to <0.1 (medium grey), 0.01 to <0.5 (light grey), <0.01 (white)]. The contour-enhanced funnel plots may suggest funnel-plot asymmetry is due to publication bias when less precise (smaller) studies are missing in the non-significant regions. Quantitative assessment of publication bias was also performed using Egger's and Begg's tests set at a significance level of p<0.1.

CI, confidence interval; HDL-C, high density lipoprotein cholesterol

Supplemental Figure S32. Publication bias funnel plots for the effect of extracted pulse proteins on TG

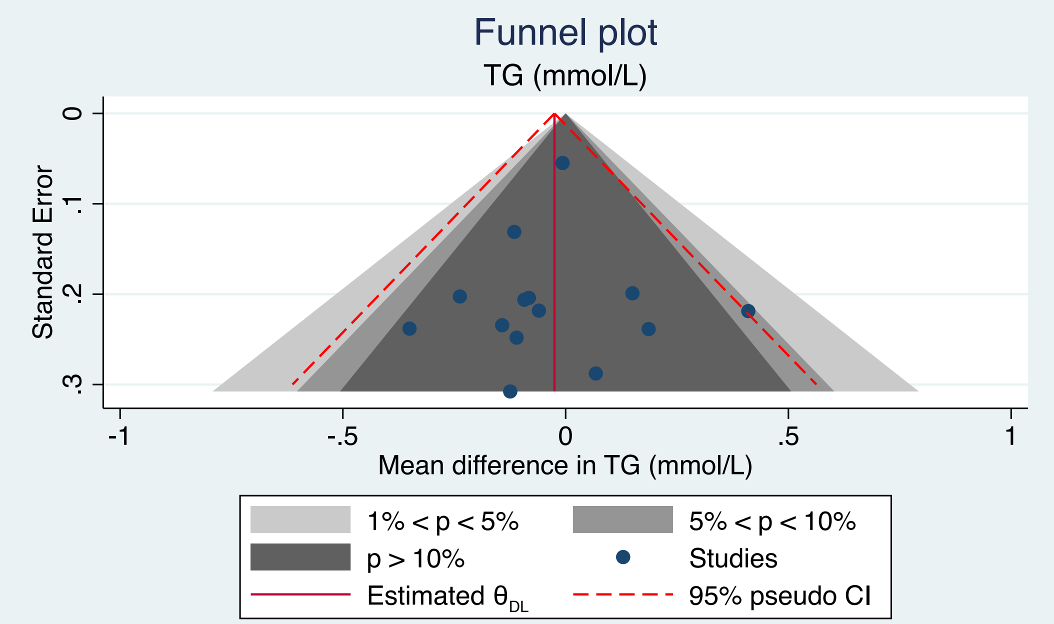

P-values
Begg's test: 1.000
Egger's test: 0.759

Contour-enhanced funnel plot is a scatterplot of each trial comparison weighted mean difference (MD) on the x-axis with the standard error (SE) representing precision on the y-axis. The vertical solid red line represents the pooled effect estimate and the dashed red lines represent the pseudo-95% confidence limits. The blue dots represent individual trial comparisons. The contour regions define the regions for the test of significance of individual study effect size for a given p-value range >0.1 (dark grey), 0.5 to <0.1 (medium grey), 0.01 to <0.5 (light grey), <0.01 (white)]. The contour-enhanced funnel plots may suggest funnel-plot asymmetry is due to publication bias when less precise (smaller) studies are missing in the non-significant regions. Quantitative assessment of publication bias was also performed using Egger's and Begg's tests set at a significance level of p<0.1.

CI, confidence interval; TG, triglyceride
